# A panel of emerging EMT genes identified in malignant mesothelioma

**DOI:** 10.1038/s41598-022-04973-x

**Published:** 2022-01-19

**Authors:** Licun Wu, Shaheer Amjad, Hana Yun, Sendurai Mani, Marc de Perrot

**Affiliations:** 1 Latner Thoracic Surgery Research Laboratories, Division of Thoracic Surgery, Toronto General Hospital Research Institute, University Health Network, University of Toronto, Toronto, Canada; 2 Toronto General Hospital and Princess Margaret Cancer Centre, University Health Network, Toronto, Canada; 3 Institute of Medical Science, University of Toronto, Toronto, Canada; 4 Department of Translational Molecular Pathology, MD Anderson Cancer Center, The University of Texas, Houston, TX USA; 5 Department of Immunology, University of Toronto, Toronto, Canada; 6 Division of Thoracic Surgery, Toronto General Hospital, 9N-961, 200 Elizabeth Street, Toronto, ON M5G 2C4 Canada

**Keywords:** Cancer, Oncology

## Abstract

Malignant mesothelioma (MESO) is a highly aggressive cancer with poor prognosis. Epithelial–mesenchymal transition (EMT) is a critical process in malignancies involved in tumor angiogenesis, progression, invasion and metastasis, immunosuppressive microenvironment and therapy resistance. However, there is a lack of specific biomarkers to identify EMT in MESO. Biphasic MESO with dual phenotypes could be an optimal model to study EMT process. Using a powerful EMTome to investigate EMT gene signature, we identified a panel of EMT genes *COL5A2, ITGAV, SPARC* and *ACTA2* in MESO. In combination with TCGA database, Timer2.0 and other resources, we observed that overexpression of these emerging genes is positively correlated with immunosuppressive infiltration, and an unfavorable factor to patient survival in MESO. The expression of these genes was confirmed in our patients and human cell lines. Our findings suggest that these genes may be novel targets for therapeutics and prognosis in MESO and other types of cancers.

## Introduction

Malignant mesothelioma (MESO) is a rare cancer associated with poor prognosis. Clonal structure in mesothelioma may be a critical prognostic indicator^1^. Histologically, MESO is divided into three major subtypes: epithelioid, biphasic and sarcomatoid. Considerable evidence has shown that biphasic and sarcomatoid subtypes are associated with worse prognosis than the epithelioid subtype^2^, most likely indicating that MESO with dual phenotypes such as biphasic mesothelioma could be an optimal model to study the epithelial-mesenchymal transition (EMT) process, a process through which epithelial cells adopt mesenchymal features^3,4^.

The EMT is crucial not only to embryonic development but also to carcinogenesis, cancer progression, invasion and metastasis^5,6^. Under normal conditions, epithelial stem cells play critical roles in tissue repair and regeneration through self-renewal and differentiation capacity. Activation of EMT process has been implicated in normal and neoplastic epithelial stem cells during tissue regeneration and repair^7–9^.

Cellular heterogeneity and plasticity generated by EMT programs may contribute to our understanding of cancer stem cell biology and cancer metastasis. However, the links between EMT and cancer cell stemness are still elusive^10^. Cancer cells can reactivate EMT programs by increasing their aggressiveness. EMT is associated with enhanced stemness to drive cancer metastasis, recurrence and therapy resistance. While undergoing EMT, most tumor cells adopt some mesenchymal features and maintain some epithelial characteristics. Mesothelioma is characterized by epithelial and mesenchymal diversity, which may make it particularly useful to study EMT. Partial EMT can drive distinct migratory properties and enhance the epithelial-mesenchymal plasticity of cancer cells^11^. Characterization of EMT can be important to determine prognosis and, also, potentially predict response to immunotherapy. Increasing evidence demonstrates that EMT is associated with changes in the tumor immune microenvironment, suggesting that EMT could become important biomarkers in the context of immunotherapy^12^.

Epithelial cells may lose their polarity and cellular adhesions to migrate and invade stroma. EMT frequently takes place at an intermediate state between epithelial and mesenchymal features^13^. EMT is often activated during cancer cell migration, invasion and metastasis. A direct link between the EMT and the gain of epithelial stem cell properties has been investigated previously^14^. Once a metastatic tumor grows, cancer cells will trigger a reverse process of mesenchymal–epithelial transition (MET)^15^. Epithelial cells may be triggered to differentiate into a multipotent stem cell-like phenotype through EMT induction. Evidence indicates that EMT could facilitate cancer stem cell pluripotency^16^. Although all EMT subpopulations behave similar to tumor-propagating cells, these subpopulations are localized differently to regulate EMT transition states^17–19^.

A variety of EMT gene signatures have been identified dependent on different cancer types^20,21^. Each individual cancer may have a particular signature; nevertheless, some cancers may share common gene signatures^22^. Yet it remains open to clarify the most representative gene signature to each type of cancer, especially mesothelioma. The EMT signature of interest from different sources might be biased towards epithelial or mesenchymal genes, due to cancer type and gene identification method. To avoid that bias, ETome selected Top50 Epithelial and Top50 Mesenchymal genes to assess how they correlated with each phenotype^23^.

In this study, we screened EMT-involved hallmark gene sets using murine mesothelioma models, explored EMT-related genes in 32 pan-cancer cohorts, and further analyzed the EMT interactome, EMT score, and correlations with patient survival and immune enrichment in TCGA database to identify EMT specific genes of importance in MESO. These genes could have implications in other tumor types as well.

## Results

### Overlaps of up-regulated genes in mesothelioma microenvironment from microarray and scRNA-Seq data sets

**Figure 1:**
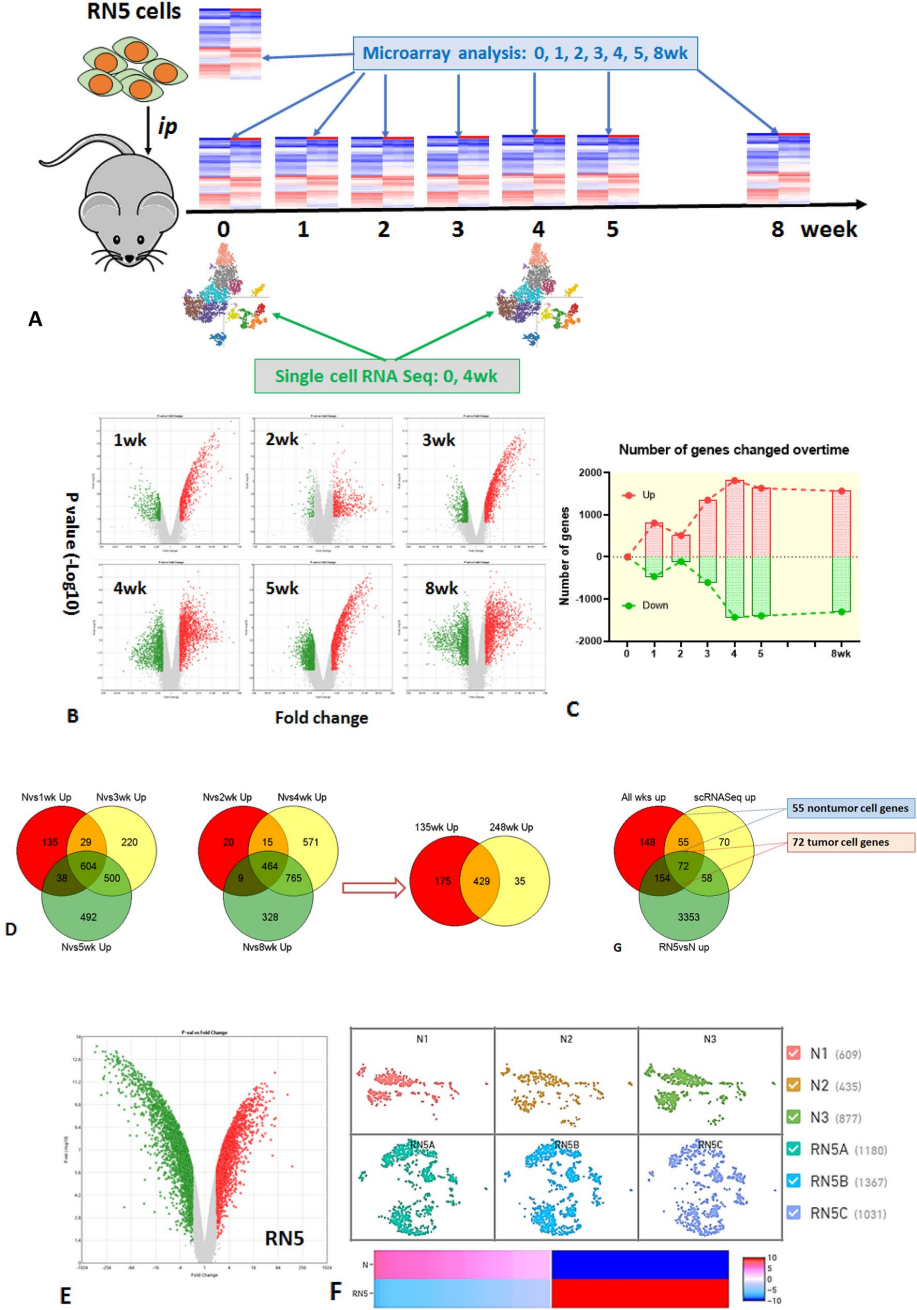
Overlaps of up-regulated genes in mesothelioma microenvironment by microarray and single cell RNA sequencing. (**A**) The schema of experimental design; (**B**) up- or down-regulated genes with significant changes over time were analyzed using microarray in scatter plots. Single cells were collected from peritoneal lavage of RN5-bearing mice at1, 2, 3, 4, 5, and 8 weeks after tumor cell *ip* injection. (**C**) Total number of genes changed over time after tumor challenge compared with naïve controls (> twofold change, p < 0.05). (**D**) The overlaps of all up-regulated genes at different time points were determined by GeneVenn and 429 common genes were found to be overexpressed in all timepoints. (**E**) Cultured mesothelioma cells RN5 were used to determine tumor genes or non-tumor genes. (**F**) Most representative time point at 4-week was selected to perform scRNA-Seq in comparison with naïve control (N) to determine up-regulated genes in tumor microenvironment. (**G**) Final overlaps of up-regulated genes derived from microarray analysis at all time points of peritoneal lavage in (**D**) (red), microarray analysis of RN5 cells with 3637 up-regulated genes in (**E**) (green) and scRNA-Seq of single cells in peritoneal lavage from tumor-bearing mice at 4 weeks with 255 up-regulated genes in (**F**) (yellow). Finally, 55 non-tumor genes and 72 tumor genes were identified by GeneVenn from the above triple data sets. Single cells or RNA from peritoneal lavage of naive mice were used as controls.

First, we screened all up-regulated genes in the mesothelioma microenvironment using peritoneal lavage. The up- or down-regulated genes with significant changes over time were analyzed using microarray from single cells collected from peritoneal lavage of RN5-bearing mice. The total number of genes changed over time after tumor challenge (> twofold change, *p* < 0.05) (Fig. 1A–C). The overlaps of all up-regulated genes at different time points were determined by GeneVenn. In total, 429 genes were found to be overexpressed at all time points compared with naïve controls (Fig. 1D). Peritoneal lavage from naïve mice (N) was used as controls.

Cultured mesothelioma RN5 cells were then used to differentiate genes originating from tumor cells (defined as “tumor genes”) from those originating from the tumor microenvironment itself (defined as “non-tumor genes”) (Fig. 1E). A total of 3637 genes were up-regulated in RN5 cells compared to the peritoneal lavage cells of naïve mice.

Single cell RNA-sequencing from the peritoneal lavage of tumor bearing mice was performed at 4 weeks. The 4-week time point was selected as the most representative time point to perform scRNA-Seq in comparison with scRNA-Seq of peritoneal lavage from naïve control since the tumor was always well established and the mice were still healthy. ScRNA-Seq demonstrated that 255 genes were up-regulated in intraperitoneal tumor microenvironment at 4 weeks of tumor-bearing mice compared to naïve mice (Fig. 1F).

The overlaps between the 429 up-regulated genes derived from the microarray analysis of peritoneal lavage, the 3637 genes from the microarray analysis of cultured RN5 cells, and the 255 genes from scRNA-Seq from peritoneal lavage single cells of tumor-bearing mice identified 55 non-tumor cell genes and 72 tumor cell genes by GeneVenn from the three data sets (Fig. 1G).

**Figure 2:**
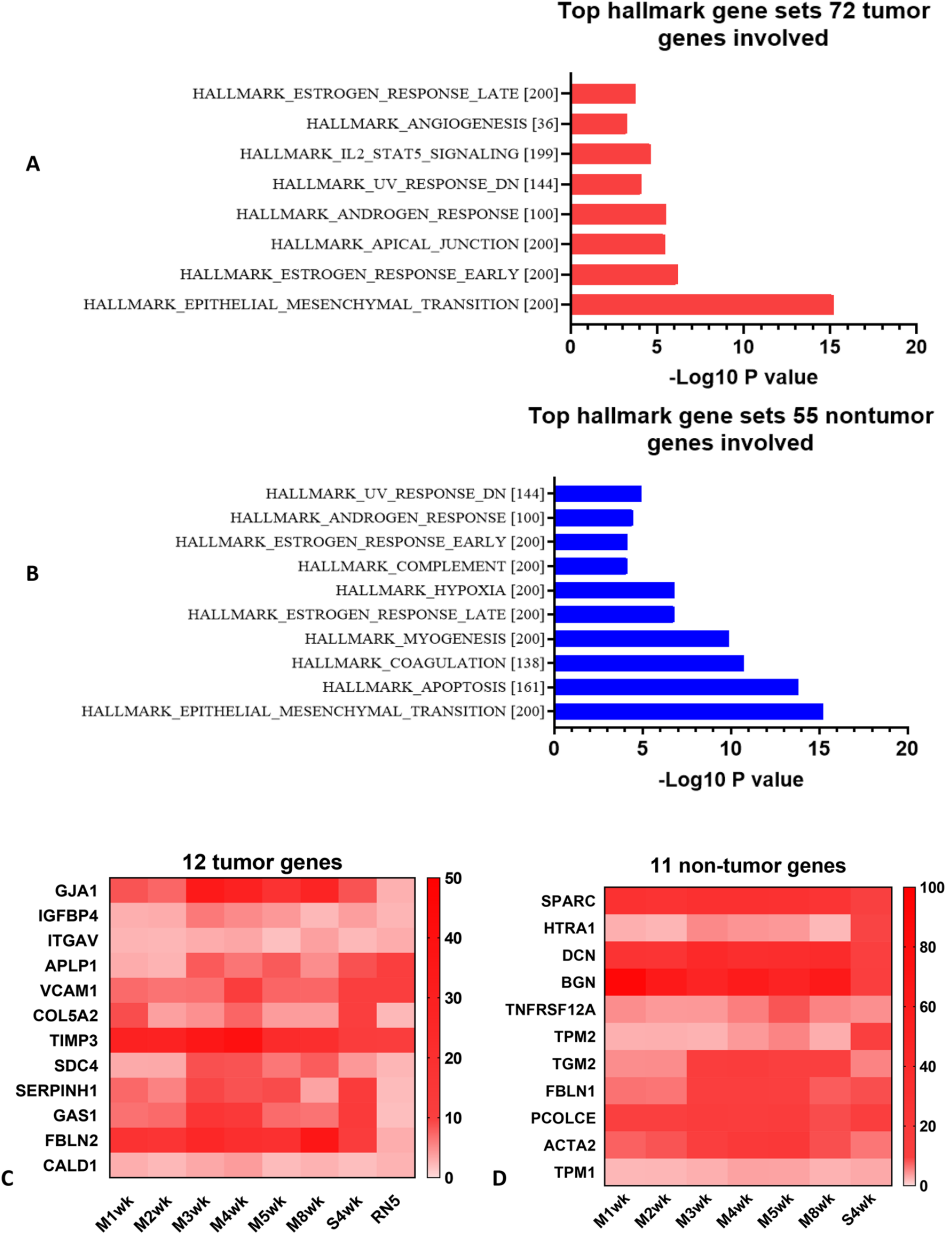
Epithelial–mesenchymal transition (EMT) is the top hallmark gene set in both tumor and non-tumor cell genes determined by GSEA. (**A**, **B**) 12/72 tumor genes and 11/55 non-tumor genes are involved in epithelial mesenchymal transition (EMT) pathway, identified by http://www.gsea-msigdb.org/gsea/msigdb. Top hallmark gene sets were presented in bar graphs. (**C**, **D**) Gene expression in heatmap: 12/72 tumor genes and 11/55 non-tumor genes. Gene set enrichment analysis (GSEA) was computed to identify the overlaps with hallmark gene sets in Molecular Signatures Database (MSigDB) Collections. with FDR q-value less than 0.05.

The selected lists of 55 non-tumor cell genes and 72 tumor cell genes are presented in “Supplementary data” (“Supplementary data”, Table 1S). The differentiation of tumor genes from non-tumor genes was confirmed in the scRNA-Seq of tumor bearing mice. The non-tumor cell genes were found to be expressed by tumor cells as well in TME (“Supplementary data”, Fig. 1S). The identified EMT genes are positively correlated each other in mRNA expression (“Supplementary data”, Fig. 2S).

### Epithelial–mesenchymal transition (EMT) is the major hallmark gene set in both tumor and non-tumor genes determined by GSEA

We then explored what hallmark pathways these selected genes are involved. Among the list of 72 tumor cell genes and 55 non-tumor cell genes, 12/72 tumor cell genes (FDR q-value 7.9E − 14, p = 1.58E − 15) and 11/55 non-tumor cell genes (FDR q-value 8.59E − 14, p = 1.72E − 15) are involved in the EMT pathway, identified by MSigDB (“Supplementary data”, Table 2S). Top hallmark gene sets are presented in bar graphs (Fig. 2A,B). Gene expression of selected lists is shown in heatmap: 12 tumor cell genes and 11 non-tumor cell genes (Fig. 2C,D). Hallmark EMT was the top pathway involved in both gene sets.

### EMTome: both gene sets were correlated with epithelial and mesenchymal phenotypes. Top genes were identified to be highly correlated with EMT signature

Since EMT is the most outstanding hallmark pathway in both gene lists, we were interested to know which genes played the most predominant roles in this process. EMT score and EMT interactome were thus performed by importing each individual gene of interest.

**Figure 3:**
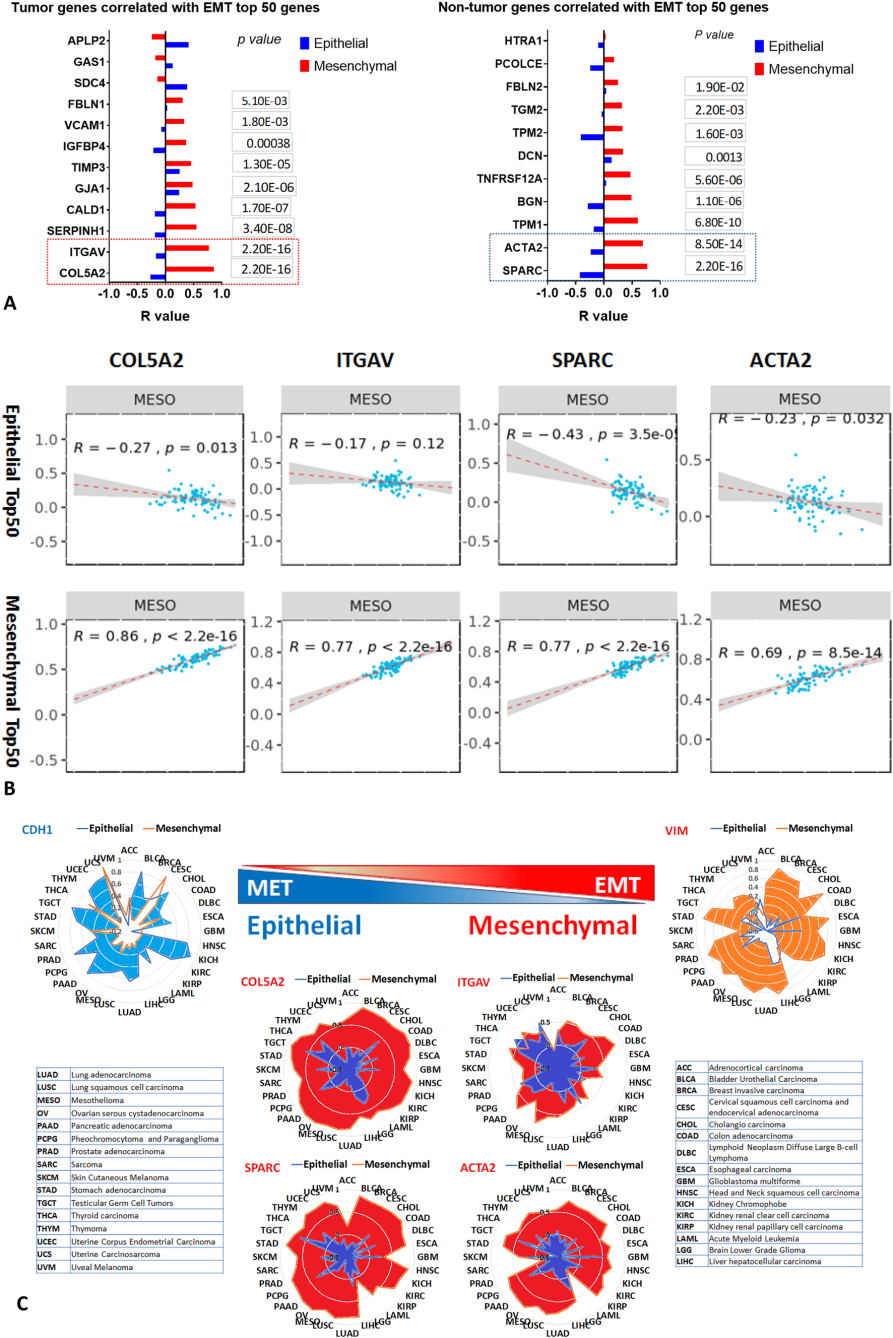
EMTome: both gene sets correlated with epithelial and mesenchymal phenotypes were analysed respectively. Top genes were identified to be highly correlated with EMT signature. EMT score and EMT interactome were performed by importing each individual gene of interest. (**A**) The correlation of each gene with Epithelial top 50 and Mesenchymal top 50 genes in pan-cancer cohorts. R scores from all genes of interest were plotted in bar graphs of both tumor and non-tumor genes, p values with mesenchymal phenotypes were also indicated. (**B**) Correlation plot from genes of interest demonstrated positive correlation with mesenchymal markers and negative correlation with epithelial markers. (**C**) Summary of 4 top selected genes correlated with EMT signature in 32 pan cancer cohorts was presented in radar graphs, and the best-known *CDH1* (Epithelial) and *VIM* (Mesenchymal) genes were included as controls. Network of OMICS profile of interest with significant correlation between gene of interest (RNA expression) in TCGA cohort MESO with OMICS profile were shown in “Supplementary data” (“Supplementary data”, Fig. 3S).

Each gene was correlated with Epithelial top 50 and Mesenchymal top 50 genes in pan-cancer cohorts. R scores from all genes of interest were plotted in bar graphs of both tumor and non-tumor genes, *p* values for the mesenchymal phenotypes were also indicated (Fig. 3A). The correlation plot demonstrated positive correlation with the mesenchymal markers and negative correlation with the epithelial markers, thus characterizing the mesenchymal phenotype (Fig. 3B). Summary of top four selected genes (*COL5A2, ITGAV, SPARC* and *ACTA2*) which were correlated with EMT signature in 32 pan cancer cohorts was presented in radar graphs, and the well-known *CDH1* (Epithelial) and *VIM* (Mesenchymal) genes were included as controls (Fig. 3C).

Network of OMICS profile of interest for the most significantly correlated genes (*COL5A2, ITGAV, SPARC* and *ACTA2*) were presented (“Supplementary data”, Fig. 3S). OMICS includes mRNA, miRNA, copy number alteration (CNA), methylation, and reverse phase protein analysis (RPPA) correlation were also analyzed. Significant correlation was considered between genes (mRNA expression) in TCGA cohort MESO with expression if FDR < 0.05.

### The mRNA expression of *COL5A2, ITGAV, SPARC* and *ACTA2* genes correlated with mesenchymal phenotype in epithelioid vs non-epithelioid (biphasic and sarcomatoid) subtypes in MESO cohort

**Figure 4:**
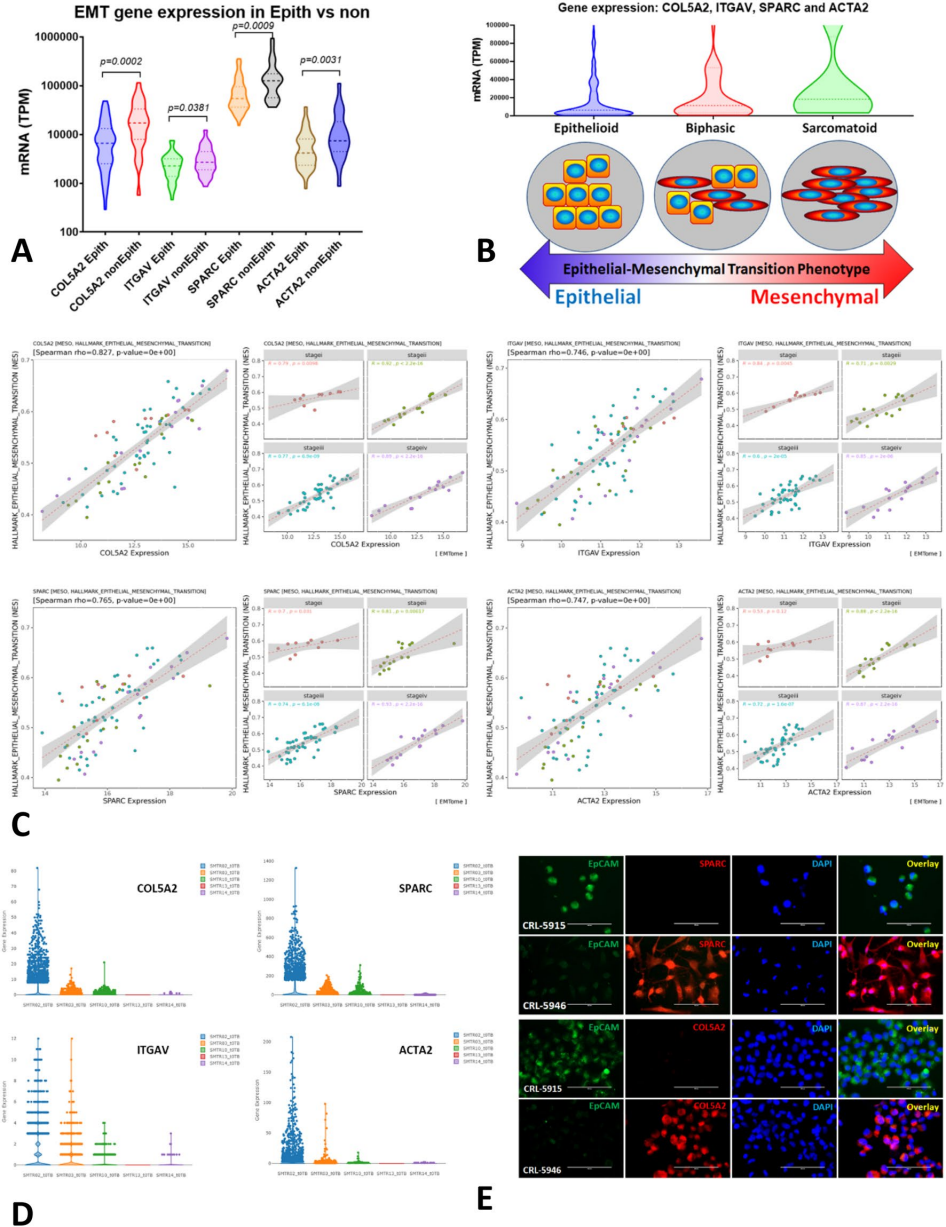
Gene expression of *COL5A2, ITGAV, SPARC* and *ACTA2* correlated with mesenchymal phenotype in Epithelioid *vs* non-Epithelioid (biphasic and sarcomatoid) subtype in TCGA MESO database, and confirmation in our patients and cell lines. (**A**) The numbers of epithelioid subtype MPM patients (n = 62), biphasic and sarcomatoid subtypes (n = 23 and n = 2, respectively) were included. Gene expression of *COL5A2, ITGAV, SPARC* and *ACTA2* (RNA expression in Transcripts Per Million-TPM) was observed to be significantly different between epithelioid and non-epithelioid subtypes in TCGA Cohort MESO (*p* < 0.05 for all comparisons). All genes had significantly higher mRNA expression in non-epithelioid than epithelioid subtypes as shown in violin graphs. (**B**) Malignant mesothelioma with dual phenotypes is likely an optimal model to study the EMT process and characterize epithelial cells changing their phenotypes into mesenchymal ones. Top graph shows the merged data of 4 genes’ expression in epithelioid, biphasic and sarcomatoid subtypes. Bottom diagram indicates the EMT plasticity is likely to mimic MESO subtypes. (**C**) In TCGA MESO cohort, gene expression of *COL5A2, ITGAV, SPARC* and *ACTA2* correlated with the hallmark EMT gene set as shown in scatter plots. The expression of each gene was positively correlated with hallmark EMT regardless of tumor stage, all *p* values for each comparison are less than 0.05. The correlation curves became more significant along with tumor progression from stage 1–4. (**D**) The scRNA-Seq data from the biopsy specimens of naïve MESO patients, the patient (SMTR02T0) with biphasic subtype had highest expression of *COL5A2, ITGAV, SPARC* and *ACTA2* genes compared with other cases who were confirmed to be epithelioid subtypes. Gene expression of *COL5A2, ITGAV, SPARC* and *ACTA2* in epithelioid vs non-epithelioid subtype in MESO patients was shown in “Supplementary data” (“Supplementary data”, Fig. 4S). (**E**) Fluorescent immunostaining of human mesothelioma cell lines for COL5A2 and SPARC, showing that sarcomatoid CRL-5946 cells expressed COL5A2 and SPARC dramatically higher compared to epithelioid CRL-5915 cells, while CRL-5915 cells expressed little for either protein but expressed epithelial marker EpCAM specifically.

Patients with epithelioid subtype (n = 62) and biphasic and sarcomatoid subtypes (n = 23 and n = 2, respectively) were included. Gene expression of *COL5A2, ITGAV, SPARC* and *ACTA2* (RNA expression in Transcripts Per Million-TPM) was significantly different between epithelioid and non-epithelioid subtypes in TCGA Cohort MESO (*p* < 0.05 for all comparisons). All four genes had significantly higher mRNA expression in non-epithelioid subtypes compared to epithelioid subtypes as shown in violin graphs (Fig. 4A).

Malignant mesothelioma therefore appears to be an optimal model to study the EMT process. The TCGA database contains only two patients with sarcomatoid MESO, thus limiting our ability to look at significant differences between biphasic and sarcomatoid MESO. Despite this limitation, the trend in mRNA expression of these four genes of interest clearly increased from epithelioid to biphasic and then sarcomatoid MESO (Fig. 4B).

In TCGA MESO cohort, gene expression of *COL5A2, ITGAV, SPARC* and *ACTA2* correlated with the hallmark EMT geneset as shown in scatter plots (Fig. 4C). The expression of each gene was positively correlated with hallmark EMT regardless of tumor stages, all *p* values for each comparison were less than 0.05, suggesting that these genes are EMT markers in MESO independent of tumor stages. It appears that along with tumor progression, q values tended to be bigger while p values tended to be smaller and far less than 0.05.

### Confirmation of EMT gene expression in human mesothelioma tissues and cell lines

In our scRNA-Seq data from the biopsy specimens of naïve MESO patients, the patient (SMTR02T0) with biphasic subtype had the highest expression of *COL5A2, ITGAV, SPARC* and *ACTA2* genes compared with other cases who were confirmed epithelioid subtypes (Fig. 4D). Dotplot was shown to compare the five cases of these four genes, and epithelial marker CDH1 and mesenchymal marker VIM were included as controls. Histology of H&E staining of biphasic (SMTR02T0) and epithelioid (SMTR03T0) subtypes were confirmed (“Supplementary data”, Fig. 4S).

Fluorescent immunostaining of human mesothelioma cell lines showed that sarcomatoid CRL-5946 cells expressed COL5A2 and SPARC dramatically higher compared to epithelioid CRL-5915 cells, while CRL-5915 cells expressed epithelial marker EpCAM specifically (Fig. 4E).

### The mRNA expression of top genes *COL5A2, ITGAV, SPARC* and *ACTA2* is associated with overall survival in epithelioid subtype and all MPM patients in TCGA database

**Figure 5:**
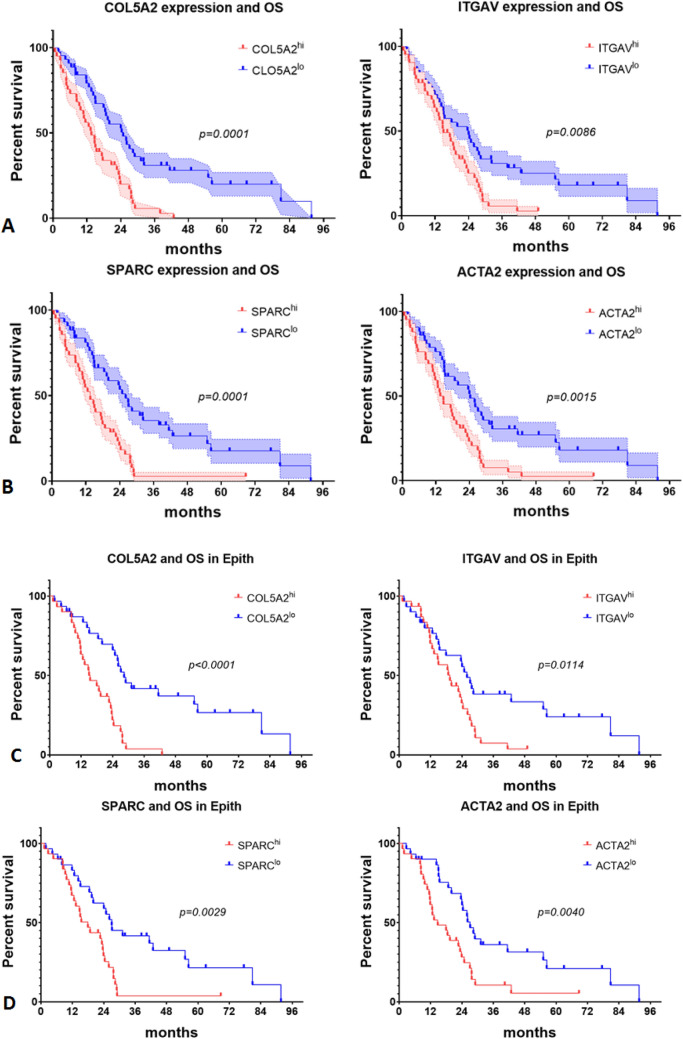
The mRNA expression of top genes *COL5A2, ITGAV, SPARC* and *ACTA2* associated with overall survival of epithelioid subtype and all MPM patients in TCGA database. (**A**) Tumor cell genes *COL5A2* and *ITGAV* expression correlated with overall survival in MESO cohort. Median survival is 13.61mon in *COL5A2*^*hi*^ and 24.89mon in *COL5A2*^*lo*^, respectively, hazard ratio: 2.318, 95% CI of ratio 1.424–3.774; Median survival is 15.02mon in *ITGAV*^*hi*^ and 24.07mon in *ITGAV*^*lo*^, respectively, hazard ratio: 1.791, 95% CI of ratio 1.116–2.875. (**B**) Non-tumor cell genes *SPARC* and *ACTA2* expression correlated with overall survival in MESO cohort. Median survival is 13.61mon in *SPARC*^*hi*^ and 26.14mon in *SPARC*^*lo*^, respectively, hazard ratio: 2.337, 95% CI of ratio 1.441–3.791; Median survival is 14.17mon in *ACTA2*^*hi*^ and 24.36mon in *ACTA2*^*lo*^, respectively, hazard ratio: 2.035, 95% CI of ratio 1.266–3.270. (**C**) Tumor genes *COL5A2* and *ITGAV* expression correlated with overall survival of epithelioid subtype. Median survival is 15.22mon in *COL5A2*^*hi*^ and 28.37mon in *COL5A2*^*lo*^, respectively, hazard ratio: 2.823, 95% CI of ratio 1.566–5.088; Median survival is 19.43mon in *ITGAV*^*hi*^ and 25.94mon in *ITGAV*^*lo*^, respectively, hazard ratio: 1.946, 95% CI of ratio 1.107–3.422. (**D**) Non-tumor genes *SPARC* and *ACTA2* expression correlated with overall survival of epithelioid subtype. Median survival is 17.95mon in *SPARC*^*hi*^ and 27.06mon in *SPARC*^*lo*^, respectively, hazard ratio: 2.179, 95% CI of ratio 1.230–3.862; Median survival is 14.76mon in *ACTA2*^*hi*^ and 27.16mon in *ACTA2*^*lo*^, respectively, hazard ratio: 2.148, 95% CI of ratio 1.213–3.803.

High expressions of tumor cell genes *COL5A2* and *ITGAV* and non-tumor cell genes *SPARC* and *ACTA2* are both positively correlated with poor overall survival and disease-free survival in MESO cohort (Fig. 5). The results indicate that overexpression of these genes of interest is unfavourable prognostic factors in mesothelioma patients.

Tumor cell genes *COL5A2* and *ITGAV* expression correlated with overall survival in MESO cohort. Median survival is 13.61mon in *COL5A2*^*hi*^ and 24.89mon in *COL5A2*^*lo*^, respectively, hazard ratio: 2.318, 95% CI of ratio 1.424–3.774; Median survival is 15.02mon in *ITGAV*^*hi*^ and 24.07mon in *ITGAV*^*lo*^, respectively, hazard ratio: 1.791, 95% CI of ratio 1.116–2.875 (Fig. 5A).

Non-tumor cell genes *SPARC* and *ACTA2* expression correlated with overall survival in MESO cohort. Median survival is 13.61mon in *SPARC*^*hi*^ and 26.14mon in *SPARC*^*lo*^, respectively, hazard ratio: 2.337, 95% CI of ratio 1.441–3.791; Median survival is 14.17mon in *ACTA2*^*hi*^ and 24.36mon in *ACTA2*^*lo*^, respectively, hazard ratio: 2.035, 95% CI of ratio 1.266–3.270 (Fig. 5B).

Tumor genes *COL5A2* and *ITGAV* expression also correlated with overall survival in the epithelioid subtype, demonstrating that epithelioid MESO can acquire EMT characteristics affecting survival before being categorized as biphasic. Median survival is 15.22mon in *COL5A2*^*hi*^ and 28.37mon in *COL5A2*^*lo*^, respectively, hazard ratio: 2.823, 95% CI of ratio 1.566–5.088; Median survival is 19.43mon in *ITGAV*^*hi*^ and 25.94mon in *ITGAV*^*lo*^, respectively, hazard ratio: 1.946, 95% CI of ratio 1.107–3.422 (Fig. 5C).

Similarly, non-tumor genes *SPARC* and *ACTA2* expression correlated with overall survival in epithelioid subtype. Median survival is 17.95mon in *SPARC*^*hi*^ and 27.06mon in *SPARC*^*lo*^, respectively, hazard ratio: 2.179, 95% CI of ratio 1.230–3.862; Median survival is 14.76mon in *ACTA2*^*hi*^ and 27.16mon in *ACTA2*^*lo*^, respectively, hazard ratio: 2.148, 95% CI of ratio 1.213–3.803 (Fig. 5D).

### Gene expression of *COL5A2, ITGAV, SPARC* and *ACTA2* correlated with immune enrichment in MESO cohort of TCGA database

**Figure 6:**
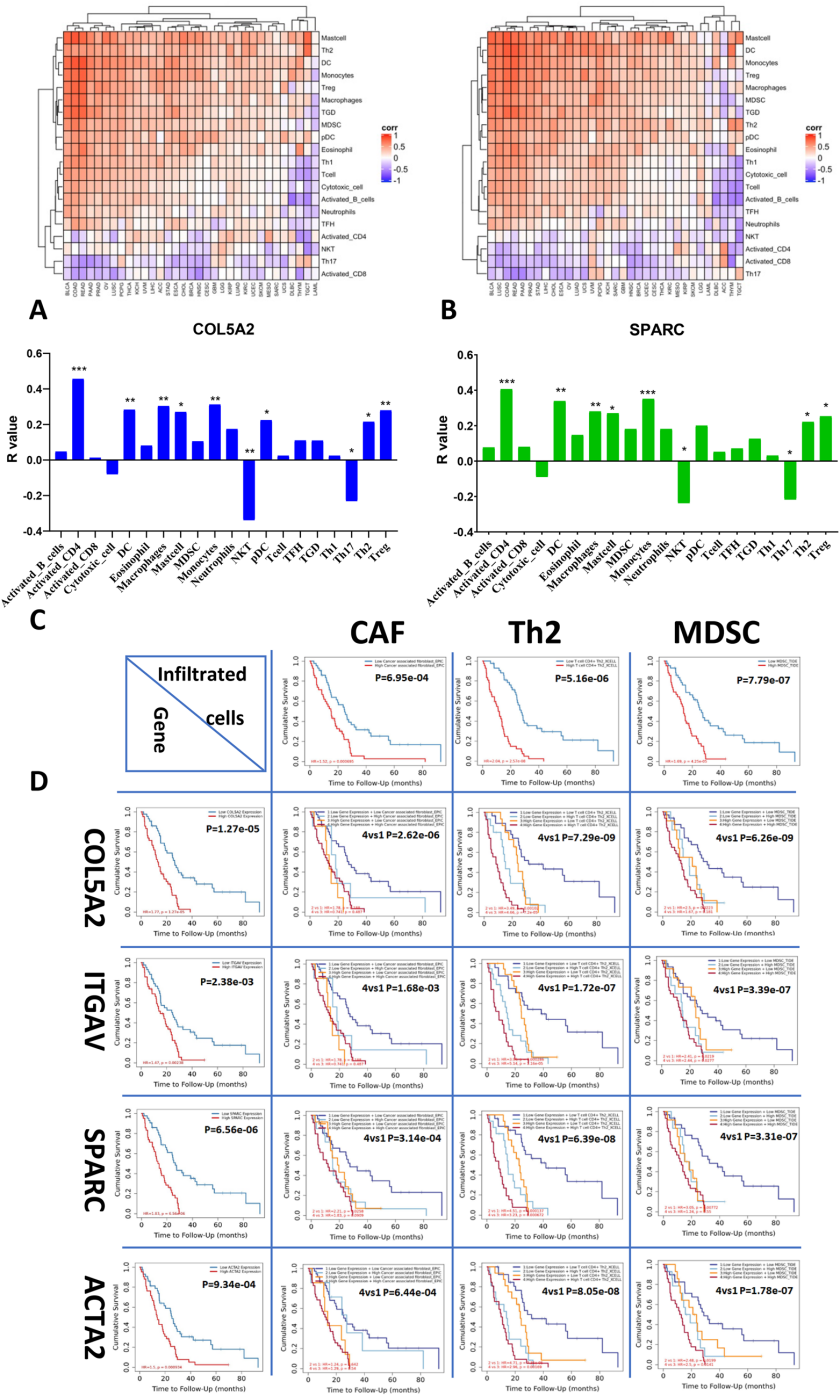
EMT gene expression and immune cell infiltration in correlation with clinical outcome in MESO cohort of TCGA database. After importing these genes into EMTome, the correlation of gene expression with the immune enrichment was analyzed in the TCGA pan cancers. *COL5A2* and *SPARC* genes had similar patterns characterized by positive correlation with the activated CD4 T cells, macrophages, monocytes, mast cells, Th2 and Treg cells, and negative correlation with Th17 and NKT cells. (**A**) Heatmaps included 32 cancers of *COL5A2*; (**B**) Heatmaps included 32 cancers of *SPARC*. (**C**) Correlation with immune enrichment in MESO cohort was shown in bar graphs of *COL5A2* and *SPARC*. The most outstanding changes of gene expression were positively correlated with immunosuppressive enrichment including macrophages, Th2, and Treg cells, while negatively correlated with immune enrichment NKT and Th17 cells. These genes were shown quite similarly (“Supplementary data”, Figs. 5S, 6S and 7S). Clinical relevance of tumor immune subsets with gene expression was explored using Timer 2.0 (**D**). The infiltration of cancer-associated fibroblasts (CAF), Th2 cells and MDSC either alone or in correlation with EMT gene expression increased the risk of MESO. Cox proportional hazard model ran Cox regression and presented clinical relevance of the normalized coefficient infiltrates and correlated with *COL5A2, ITGAV, SPARC* and *ACTA2* gene expression. Kaplan–Meier curves display gene expression associated with clinical outcome in MESO cohort, corresponding to each gene of interest and immune infiltrates of CAF, Th2, and MDSC, and M2 macrophage and Treg cells (“Supplementary data”, Fig. 9S). For all integrative survival curves of gene expression and immune enrichment: 1. Blue curve: Low gene expression + Low immune enrichment; 2. Light blue curve: Low gene expression + High immune enrichment; 3. Orange curve: High gene expression + Low immune enrichment; 4. Red curve: High gene expression + High immune enrichment.

To assess the correlation of gene expression with the immune enrichment by EMTome, we imported these genes into the TCGA pan cancers. *COL5A2, ITGAV, SPARC* and *ACTA2* genes had similar patterns in positive correlation with activated CD4 T cells, macrophages, monocytes, mast cells, Th2 and Treg cells, while these genes had negative correlation with Th17 and NKT cells. The representative genes *COL5A2* and *SPARC* were presented in Fig. 6. Heatmaps included 32 cancers (*COL5A2* in Fig. 6A; *SPARC* in Fig. 6B). Correlation with immune enrichment in MESO cohort was shown in bar graphs (Fig. 6C). The most outstanding changes of gene expression were positively correlated with immunosuppressive enrichment including macrophages, Th2, and Treg cells, while negatively correlated with immune enrichment NKT and Th17 cells. These genes were shown quite similarly (“Supplementary data”, Figs. 5S, 6S and 7S).

More strikingly, Timer 2.0 gene module showed strong positive correlation between gene expression and cancer associated fibroblasts (CAF), immunosuppressive infiltrates of MDSC and Th2 cells in MESO (“Supplementary data”, Fig. 8S). Clinical relevance of tumor immune subsets with gene expression showed that either the infiltration of CAF, Th2 cells or EMT gene expression was significantly associated with survival of MESO cohort. Dual high levels of immunosuppressive infiltration and EMT gene expression dramatically increased the risk of poor prognosis, compared to dual low variables. Cox proportional hazard model ran Cox regression and presented clinical relevance of the normalized coefficient infiltrates and correlated with *COL5A2, ITGAV, SPARC* and *ACTA2* gene expression. Kaplan–Meier curves display gene expression associated with clinical outcome in MESO cohort, corresponding to each gene of interest and immune infiltrates of CAF, Th2, and MDSC (Fig. 6D), and M2 macrophage and Treg cells (“Supplementary data”, Fig. 9S).

## Discussion

Our previous studies and that of others have indicated that non-epithelioid mesothelioma cells are more aggressive and have higher capacity to form mesospheres in vitro and in vivo^24,25^. Compared with epithelioid subtype, non-epithelioid mesothelioma cells were more resistant to chemoradiation, and the surviving cells had enhanced stemness^26^. Our findings in this study imply that EMT may be a critical process to predict cancer cell invasiveness and stemness. Nowadays the linkage between the EMT and cancer cell stemness, metastasis and resistance to therapy has been driving efforts to develop novel therapeutic targets^27^.

EMT occurs through distinct intermediate states. Biphasic mesothelioma characterized by dual histology could thus best mimic the intermediate hybrid state of EMT stage while transitioning from epithelial to partially and completely mesenchymal states. A recent study revealed that in mouse and human squamous cell carcinoma, loss of function of FAT1 could promote tumor initiation, progression, invasion, stemness and metastasis through inducing a hybrid EMT state^28^. EMT genes can be novel potential targets to interrupt cancer invasion and metastasis^29^. EMT is driven by the SNAIL and ZEB transcriptional repressors of epithelial genes. TGF-β is a potent inducer of EMT specifically working with RAS-MAPK signaling pathway^30^.

Our findings identify previously less well recognized genes that promote EMT in MESO. Reversal of EMT into MET process may reduce the capacity of invasiveness, cancer cell stemness and metastasis of mesothelioma and augment their sensitivity to therapy. The genes *COL5A2, ITGAV, SPARC* and *ACTA2* play critical roles resulting in EMT in MESO. Therefore, these genes may be potential therapeutic targets as well as prognostic indicators in mesothelioma.

A previous study investigating the relationship between collagen type V alpha 2 chain (*COL5A2*) expression and clinical outcome in bladder cancer patients observed that patients with lower expression of COL5A2 had better survival than those with higher expression^31^. Recently, a study evaluated the mutational profile of a panel of 34 genes including COL5A2 gene in MESO. They found that *BAP1* mutation was related to a prolonged survival of patients treated with platinum/pemetrexed regimens^32^. Mutations in COL5A2 was observed in 6% of the patients. However, the expression level of COL5A2 was not analyzed and therefore its impact on outcome could not be directly addressed.

It is well known that integrin signaling drives multiple cancer cell functions, including tumor initiation, epithelial plasticity, metastasis, and resistance to targeted therapies^33^. Integrin alpha V (*ITGAV*), a transmembrane glycoprotein, has been found to enhance tumor progression. *ITGAV* expression is associated with shortened overall survival in esophageal adenocarcinoma^34^. Genes *ITGAV, FN1*, and *ITGB1* were shown to be the targets of miR-9-3p, which could inhibit proliferation and metastases of nasopharyngeal carcinoma by downregulating *FN1, ITGB1*, and *ITGAV*, thus inhibiting the EMT process^35^.

*SPARC* (Secreted protein acidic and rich in cysteine) gene was up-regulated specifically at the early stage of lung adenocarcinoma consistently with TCGA transcriptome database when EMT markers were screened in a cellular model and validated in lung adenocarcinoma^36^. There was a proteomics-based study to identify SPARC as a prognostic biomarker in MESO. They found that high level of circulating SPARC was associated with poor prognosis^37^. However, no study could be found in mesothelioma.

*ACTA2* gene encodes α-smooth muscle actin (αSMA) protein, which has been shown to be a hallmark of EMT in lens epithelial cells. The increased level of histone H4 acetylation at the *ACTA2* promoter region was associated with EMT of lens epithelial cells^38^.

Epithelioid mesothelioma is known to have a better prognosis in comparison with sarcomatoid and biphasic types. However, within patients with epithelioid subtype, there is a lack of good prognostic markers even though some tumors are more aggressive than others. Nuclear atypia could have an impact on survival in epithelioid mesothelioma, and nuclear grade was confirmed to correlate with survival in a multi-institutional study^39,40^, but further studies will be required to demonstrate the validity of this marker in clinical practice.

According to a recent study, the prognosis of epithelioid mesothelioma patients is impacted by a protein called connective tissue growth factor (CTGF). CTGF, a secreted protein produced by both mesothelioma cells and cancer-associated fibroblasts, can promote mesothelioma cell invasion. Patients with lower levels of this protein had a better prognosis and longer survival. Using surgical specimens of epithelioid MPM, the authors evaluated the clinicopathological significance of αSMA expression, the most widely used marker of CAFs, the expression of CTGF, and the extent of fibrosis by immunohistochemistry. They showed that the expression of αSMA and CTGF in CAFs correlated with poor prognosis in epithelioid subtype MESO patients^41^.

Overexpression of the emerging EMT genes had positive correlation with the activated CD4 T cells, macrophages, monocytes, mast cells, Th2 and Treg cells, suggesting that these genes may play critical roles in modulating the immunosuppressive microenvironment. In contrast, negative correlation was observed with NKT cells, supporting a notion that EMT process may abrogate the immune response against tumor. Previous evidence showed that EMT transcriptional factors lead to immunosuppressive cell infiltration, resulting in a tumor immunosuppressive microenvironment. The immunosuppressive cells in turn promote EMT in tumor cells. The interaction between EMT and immunosuppression promotes tumor progression^42^. Tumor-infiltrating immune cells release a wide variety of inflammatory mediators and growth factors to facilitate immunosuppressive microenvironment and EMT plasticity. The immunosuppressive TME is a critical hurdle to the efficacy of cancer immunotherapy, therefore, targeting EMT could improve the outcome of immunotherapy by restricting its immunosuppressive impact^43–45^.

Our study may be the first time to demonstrate that strong positive correlation of panel EMT genes overexpression with poor clinical outcome, and immunosuppressive enrichment in MESO, indicating that these newly identified genes are most likely powerful drivers of EMT process in MESO. Therefore, regulation of each individual gene expression and its signalling pathways might potentially control EMT process thus leading to development of novel strategies for MESO therapeutics. SPARC protein has been observed upregulated in MESO cell lines^46^. Transcriptomic studies have identified that EMT-linked genes may contribute towards the resistance of chemotherapy and immunotherapy, suggesting that targeting EMT genes may be potentially applied to treat MESO patients^47^. This work has important implications for targeting nonimmune components in TME to improve the efficacy of chemotherapy and boost the responses to immunotherapy of cancer patients.

In conclusion, utilising transcriptomic and EMTome analysis, we identified these EMT genes that are overexpressed in tumor microenvironment of mouse and human mesothelioma. These genes strongly correlate with an EMT signature and prognosis in MESO. Besides a few sporadic studies, this may be the first study to identify *COL5A2, ITGAV SPARC and ACTA2* as a panel of emerging EMT genes in mesothelioma. Furthermore, these genes may be universal EMT markers for most cancer types, as well as prognostic indicators for overall survival across cancer types. Due to a lack of biomarkers to predict survival in epithelioid subtype, it is difficult to further stratify this subgroup of MESO. Encouragingly, this study demonstrated that overexpression of these four genes in epithelioid mesothelioma was associated with poor prognosis, indicating that these genes could potentially serve as independent prognostic indicators in this subtype of mesothelioma.

More importantly, overexpression of these genes is associated with an immunosuppressive microenvironment promoted by the EMT process. Therefore, these genes could be potential biomarkers and targets to select appropriate immunotherapy.

## Materials and methods

The schema of experimental design was depicted in Fig. 1A. Briefly, murine mesothelioma cells were injected intraperitoneally (*ip*) into wild-type mice, and peritoneal lavage was collected at different time points to investigate gene expression profile in tumor microenvironment (TME). Naïve mice, and cultured tumor cells were included as controls. Microarray and single cell RNA sequencing (scRNA-Seq) were performed at indicated times.

### Murine mesothelioma cells and mice

The murine mesothelioma cell line RN5 derived from C57BL/6 mice was established recently by our team and presents characteristics of biphasic mesothelioma^48,49^. Cells were maintained in RPMI1640 medium supplemented with 10% fetal bovine serum and 1% penicillin and streptomycin at 37 °C in an atmosphere containing 5% CO_2_. Cells were treated with prophylactic 5 µg/ml Plasmocin™ (Invivogen) for at least 2 weeks and were confirmed as mycoplasma-free. Exponentially growing cells (approximately 90% confluence) were prepared and injected into the 6–8 week-old C57BL/6 mice purchased from the Jackson Laboratories.

RN5 cells (2 × 10^6^/200 µl PBS) were injected *ip* into 40 female mice, and five mice were sacrificed once weekly up to 8 weeks, including naive mice.

### RNA extraction for microarray

Peritoneal lavage was collected by rinsing with 5 ml of PBSF and single cells were prepared by removing tumor spheroids with a cell strainer (Ø40µm). The procedure of RNA extraction was performed according to the manufacturer’s instruction (QIAGEN, RNeasy Microarray Tissue Mini Kit, CA). Total RNA was treated with a Purelink DNase set (Thermo Fisher Sci., CA). Gene expression profile was evaluated by Affymetrix microarray assay by the Genomic Centre of the Hospital for Sick Children, Toronto, Canada. Array Type: MoGene-2_0-st; Analysis Type: Expression (Gene); Analysis Version: version 1; Genome Version: mm10 (*Mus musculus*); Annotation: MoGene-2_0-st-v1.na36.mm10.transcript.csv.

Microarray data were analyzed using a software Transcriptome Analysis Consortium (TAC Version4.0) provided by Affymetrix Inc., Applied Biosystems, ThermoFisher Scientific (Santa Clara, CA).

### Patients with mesothelioma

Five naïve patients were confirmed with a diagnosis of malignant pleural mesothelioma. Tumor biopsy tissues were processed freshly for scRNA-seq analysis. This study was approved by our institution (REB#19-5858) and all patients signed the consent forms.

### Single cell RNA sequencing (scRNA-Seq)

Fresh single cells obtained from peritoneal lavage were processed by Princess Margaret Genomic Centre, University Health Network (UHN), following the standard protocol (www.pmgenomics.ca). Loupe Cell Browser v5.0.0 provided by 10× Genomics was used to analyze single cell gene expression in clusters. A reference mouse genome (mm10) was selected to set threshold of mitochondrial UMIs as over-expression of mitochondrial genes could signify poor quality or dying cells. Globally distinguishing genes were moved for further analysis.

Patient tumor samples were collected from the outpatient biopsy. This study was approved by UHN Research Ethics Board (REB#19-5858) and all patients signed the consent form.

### Analytical tools for our data sets

The online analytical tools such as Transcriptome Analysis Console (TAC) Software, Loupe Cell browser https://support.10xgenomics.com/single-cell-gene-expression, https://crescent.cloud/, Gene set enrichment analysis (GSEA) http://www.gsea-msigdb.org/gsea/msigdb/index.jsp, GeneVenn http://genevenn.sourceforge.net/. The Cancer Genome Atlas (TCGA) https://www.cbioportal.org/, EMTome http://www.emtome.org/, and Timer2.0 http://timer.comp-genomics.org/ were employed to identify the EMT genes and signatures in correlation with clinical outcome in mesothelioma.

### Overlaps of all genes with significant change

After transcriptomic analysis with TAC and Loupe Cell, the overlaps of all gene lists were determined by GeneVenn. The final overlaps were compared by three major gene lists: all time points up-regulated genes (All wks up), culture RN5 cells up-regulated genes (RN5vsN up) and scRNA-Seq up-regulated genes at 4 weeks (scRNASeq up).

### To compute hallmark genesets for selected genes

By importing the gene list from each comparison, GSEA was able to compute and identify the hallmark gene sets in Molecular Signatures Database (MSigDB) Collections. The cut-off value was selected with FDR q-value of 0.05.

### EMT genes and signatures of interest analyzed by EMTome

This online program was developed by Dr. S. Mani Team, MD Anderson Cancer Center, Houston TX. The database EMTome collected EMT and MET core gene signatures from publications to identify their interaction with miRNA, transcription factors and proteins using cell lines (CCLE), TCGA, and other datasets. The EMTome acts as resource and a platform to interrogate EMT signatures across cancer types^50^.

### Fluorescent immunostaining in human mesothelioma cell lines

Human mesothelioma cell lines CRL-5915 (characterized with more epithelioid) and CRL-5946 (more sarcomatoid), which were provided by ATCC, were used to stain COL5A2 and SPARC proteins in cultured cells. Cells were fixed with 1% PFA for 15 min, and 1% BSA was to block endogenous enzymes. Triton X-100 (0.25%, 5 min) was added for permeabilization. Primary antibodies rabbit polyclonal to COL5A2 (ab134800) and SPARC (ab14174) were used 1:100 for 1 h at RT, and the secondary antibody anti-rabbit IgG1 conjugated with Alexa-555 was 1:1000 for 1 h at RT. Anti-human EpCAM-FITC monoclonal antibody was applied 1:100. Mount medium containing DAPI was applied to display nuclei.

### Analysis of immune infiltrates, gene expression and clinical outcome in MESO by Timer 2.0

TIMER is a comprehensive resource for systematic analysis and visualization functions of tumor infiltrating immune cells, and their association with gene expression and clinical outcome (http://timer.cistrome.org/)^51^.

### Statistical analysis

Transcriptomic analysis was made according to the filter criteria: Fold Change > 2 or < − 2, P < 0.05. The numbers of genes are differentially expressed genes that pass through the filter criteria.

Gene expression (mRNA in TPM) in the violin graph was plotted using Prism 8.0. Unpaired two-tailed *t* test, with a p-value less than 0.05 was considered a significant difference.

Significant correlation was considered between genes (mRNA expression) in TCGA cohort MESO with expression if false discovery rate (FDR) < 0.05.

Log-rank (Montel–Cox) test was used to compare survival curves. A *p-*value less than 0.05 was considered a significant difference. Group cutoff median was selected to determine the cutoff-high (50%) and cutoff-low (50%) of gene expression, approximately 50%. Hazard ratio was calculated based on Cox PH Model with 95% Confidence Interval (95% CI).

### Ethics approval and informed consent of participants

At University Health Network (UHN), research involving humans conducted within the jurisdiction or under the auspices of UHN must be reviewed and granted written approval by the UHN Research Ethics Board (REB) prior to commencement of research (https://www.uhnresearch.ca/). This applies to research involving humans that is conducted by a UHN Principal Investigator (PI). This study was approved by the Research Ethics Board (REB Approval No. 19-5858) of UHN and performed in accordance with the Declaration of Helsinki, and informed consent from the patients was signed prior to the study.

## Supplementary Information

Supplementary Information.

## Data Availability

The raw data of microarray and scRNA-Seq are available and ready to be uploaded to the public structured data depository. All data are available upon request to Dr. Marc de Perrot.
