# Supplementary material for: A panel of emerging EMT genes identified in malignant mesothelioma
Source: Sci Rep. 2022 Jan 19;12:1007. doi: 10.1038/s41598-022-04973-x (PMC8770645; doi:10.1038/s41598-022-04973-x)

Supplementary data

Table 1S Identification of EMT hallmark gene sets. Tumor cell genes (top) and non-tumor cell genes (bottom) by Gene Set Enrichment Analysis-Molecular Signatures Database (GSEA-MSigDB)

12 tumor cell genes overlapped in EMT hallmark gene set

**Symbol**	**Gene Description**
GJA1	gap junction protein alpha 1 [Source:HGNC Symbol;Acc:HGNC:4274]
IGFBP4	insulin like growth factor binding protein 4 [Source:HGNC Symbol;Acc:HGNC:5473]
ITGAV	integrin subunit alpha V [Source:HGNC Symbol;Acc:HGNC:6150]
APLP1	amyloid beta precursor like protein 1 [Source:HGNC Symbol;Acc:HGNC:597]
VCAM1	vascular cell adhesion molecule 1 [Source:HGNC Symbol;Acc:HGNC:12663]
COL5A2	collagen type V alpha 2 chain [Source:HGNC Symbol;Acc:HGNC:2210]
TIMP3	TIMP metallopeptidase inhibitor 3 [Source:HGNC Symbol;Acc:HGNC:11822]
SDC4	syndecan 4 [Source:HGNC Symbol;Acc:HGNC:10661]
SERPINH1	serpin family H member 1 [Source:HGNC Symbol;Acc:HGNC:1546]
GAS1	growth arrest specific 1 [Source:HGNC Symbol;Acc:HGNC:4165]
FBLN2	fibulin 2 [Source:HGNC Symbol;Acc:HGNC:3601]
CALD1	caldesmon 1 [Source:HGNC Symbol;Acc:HGNC:1441]

11 non-tumor cell genes overlapped in EMT hallmark gene set

**Symbol**	**Gene Description**
SPARC	secreted protein acidic and cysteine rich [Source:HGNC Symbol;Acc:HGNC:11219]
HTRA1	HtrA serine peptidase 1 [Source:HGNC Symbol;Acc:HGNC:9476]
DCN	decorin [Source:HGNC Symbol;Acc:HGNC:2705]
BGN	biglycan [Source:HGNC Symbol;Acc:HGNC:1044]
TNFRSF12A	TNF receptor superfamily member 12A [Source:HGNC Symbol;Acc:HGNC:18152]
TPM2	tropomyosin 2 [Source:HGNC Symbol;Acc:HGNC:12011]
TGM2	transglutaminase 2 [Source:HGNC Symbol;Acc:HGNC:11778]
FBLN1	fibulin 1 [Source:HGNC Symbol;Acc:HGNC:3600]
PCOLCE	procollagen C-endopeptidase enhancer [Source:HGNC Symbol;Acc:HGNC:8738]
ACTA2	"actin alpha 2, smooth muscle [Source:HGNC Symbol;Acc:HGNC:130]"
TPM1	tropomyosin 1 [Source:HGNC Symbol;Acc:HGNC:12010]

Supplementary data

***Figure 1S EMT genes* expressed predominantly on tumor cells**

A. Mouse data: scRNA-Seq. EMT genes expressed in tumor cell clusters.

B. Patient data: scRNA-Seq. EMT genes expressed in tumor cell clusters (MSLN+WT1+).

A

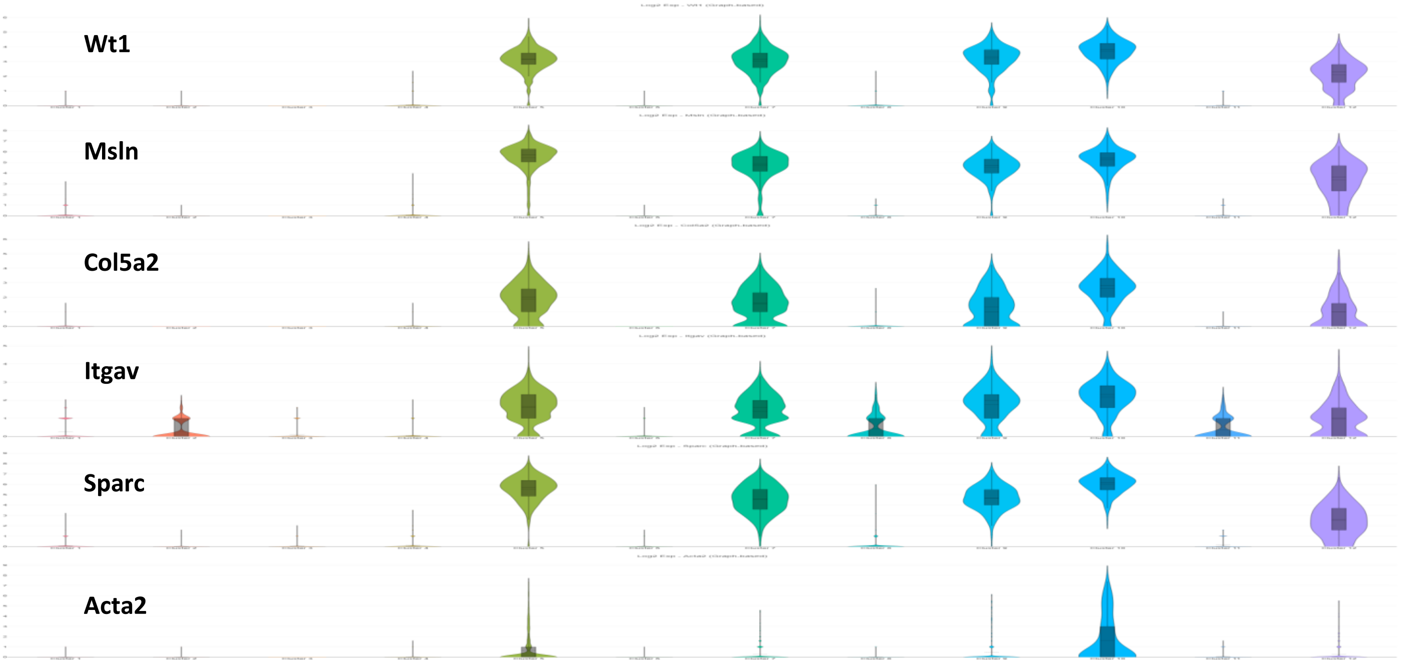

B

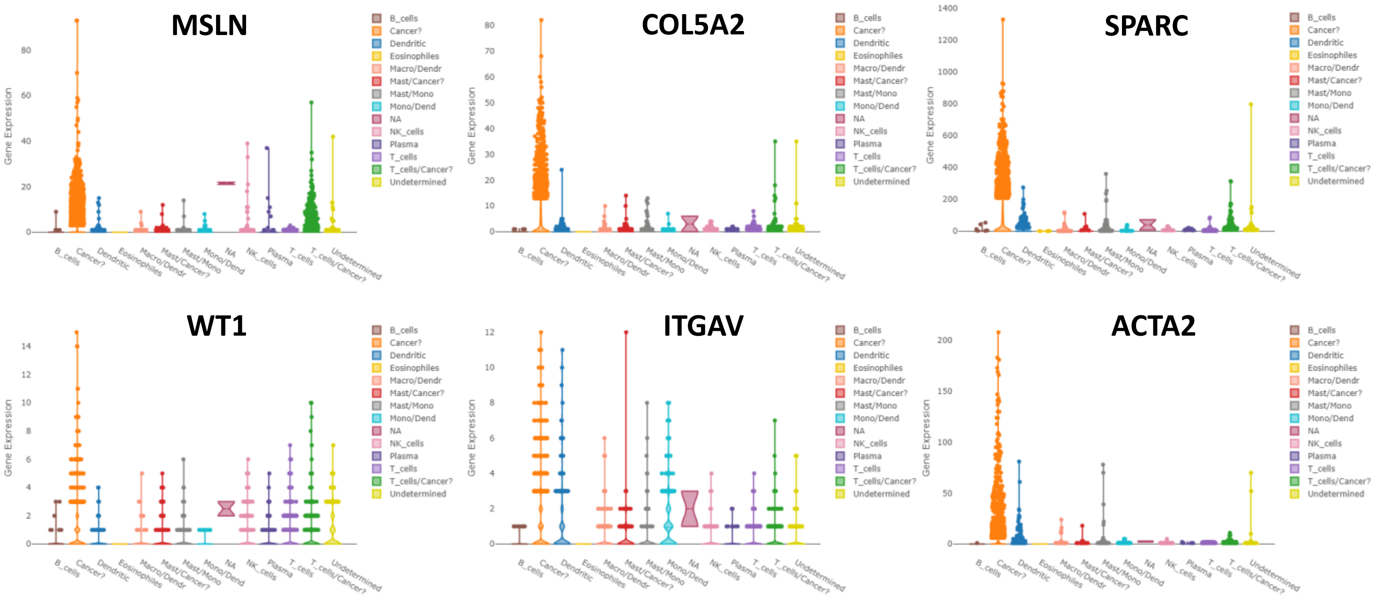

Supplementary data

Figure 2S **Co-expression of EMT genes in MESO cohort of TCGA analysed by Timer2.0.** Gene expression of *COL5A2*, *ITGAV*, *SPARC* and *ACTA2* was observed positively correlated between each other.

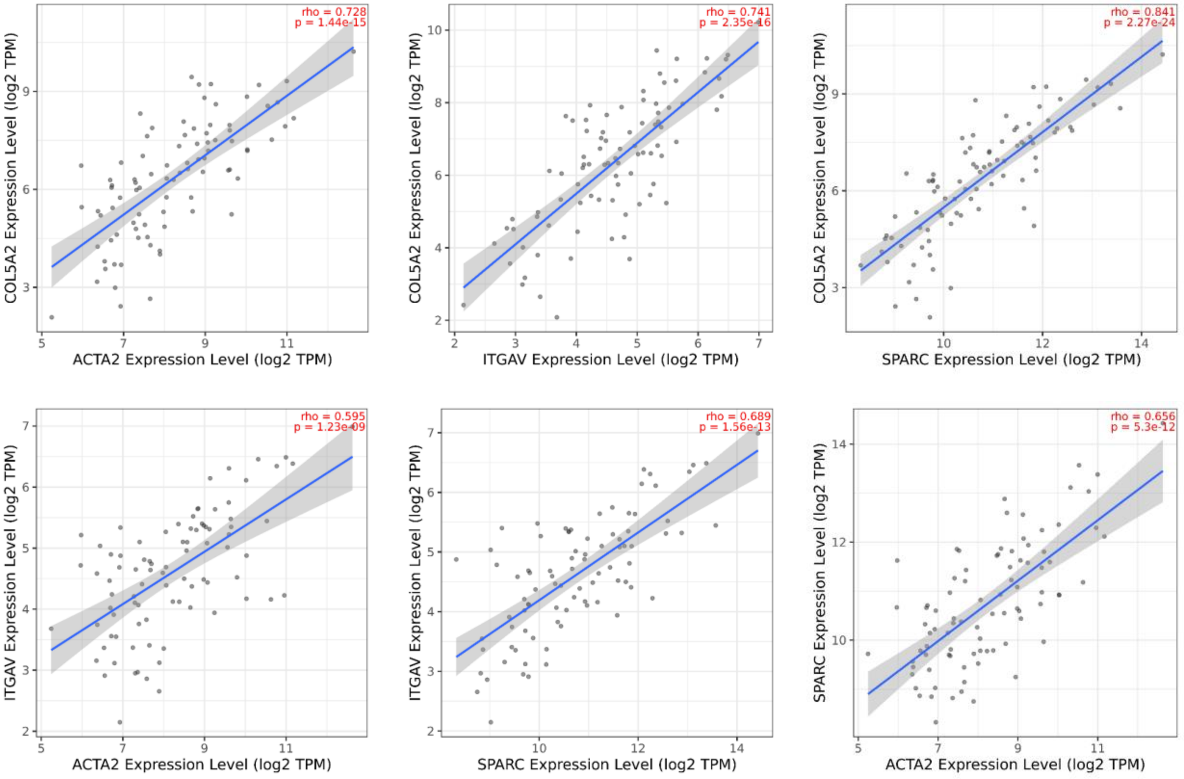

Supplementary data

Table 2S Identification of EMT hallmark gene sets. Among the list, 12/72 tumor cell genes are involved in EMT pathway.

Gene Id	Gene Symbol	Gene Description (EMT genes are highlighted)
2697	GJA1	gap junction protein alpha 1 [Source:HGNC Symbol;Acc:HGNC:4274]
3487	IGFBP4	insulin like growth factor binding protein 4 [Source:HGNC Symbol;Acc:HGNC:5473]
3685	ITGAV	integrin subunit alpha V [Source:HGNC Symbol;Acc:HGNC:6150]
333	APLP1	amyloid beta precursor like protein 1 [Source:HGNC Symbol;Acc:HGNC:597]
7412	VCAM1	vascular cell adhesion molecule 1 [Source:HGNC Symbol;Acc:HGNC:12663]
1290	COL5A2	collagen type V alpha 2 chain [Source:HGNC Symbol;Acc:HGNC:2210]
7078	TIMP3	TIMP metallopeptidase inhibitor 3 [Source:HGNC Symbol;Acc:HGNC:11822]
6385	SDC4	syndecan 4 [Source:HGNC Symbol;Acc:HGNC:10661]
871	SERPINH1	serpin family H member 1 [Source:HGNC Symbol;Acc:HGNC:1546]
2619	GAS1	growth arrest specific 1 [Source:HGNC Symbol;Acc:HGNC:4165]
2199	FBLN2	fibulin 2 [Source:HGNC Symbol;Acc:HGNC:3601]
800	CALD1	caldesmon 1 [Source:HGNC Symbol;Acc:HGNC:1441]
3880	KRT19	keratin 19 [Source:HGNC Symbol;Acc:HGNC:6436]
9022	CLIC3	chloride intracellular channel 3 [Source:HGNC Symbol;Acc:HGNC:2064]
3875	KRT18	keratin 18 [Source:HGNC Symbol;Acc:HGNC:6430]
5420	PODXL	podocalyxin like [Source:HGNC Symbol;Acc:HGNC:9171]
10397	NDRG1	N-myc downstream regulated 1 [Source:HGNC Symbol;Acc:HGNC:7679]
5269	SERPINB6	serpin family B member 6 [Source:HGNC Symbol;Acc:HGNC:8950]
79971	WLS	Wnt ligand secretion mediator [Source:HGNC Symbol;Acc:HGNC:30238]
87	ACTN1	actinin alpha 1 [Source:HGNC Symbol;Acc:HGNC:163]
24146	CLDN15	claudin 15 [Source:HGNC Symbol;Acc:HGNC:2036]
3675	ITGA3	integrin subunit alpha 3 [Source:HGNC Symbol;Acc:HGNC:6139]
55742	PARVA	parvin alpha [Source:HGNC Symbol;Acc:HGNC:14652]
4131	MAP1B	microtubule associated protein 1B [Source:HGNC Symbol;Acc:HGNC:6836]
50937	CDON	"cell adhesion associated, oncogene regulated [Source:HGNC:17104]"
216	ALDH1A1	aldehyde dehydrogenase 1 family member A1 [Source:HGNC:402]
10512	SEMA3C	semaphorin 3C [Source:HGNC Symbol;Acc:HGNC:10725]
1465	CSRP1	cysteine and glycine rich protein 1 [Source:HGNC Symbol;Acc:HGNC:2469]
4071	TM4SF1	transmembrane 4 L six family member 1 [Source:HGNC Symbol;Acc:HGNC:11853]
29785	CYP2S1	cytochrome P450 family 2 subfamily S member 1 [Source:HGNC:15654]
23468	CBX5	chromobox 5 [Source:HGNC Symbol;Acc:HGNC:1555]
4212	MEIS2	Meis homeobox 2 [Source:HGNC Symbol;Acc:HGNC:7001]
10630	PDPN	podoplanin [Source:HGNC Symbol;Acc:HGNC:29602]
105375355	UPK3B	uroplakin 3B [Source:HGNC Symbol;Acc:HGNC:21444]
90865	IL33	interleukin 33 [Source:HGNC Symbol;Acc:HGNC:16028]
22808	MRAS	muscle RAS oncogene homolog [Source:HGNC Symbol;Acc:HGNC:7227]
10232	MSLN	mesothelin [Source:HGNC Symbol;Acc:HGNC:7371]
10417	SPON2	spondin 2 [Source:HGNC Symbol;Acc:HGNC:11253]
128414	NKAIN4	sodium/potassium transporting ATPase interacting 4 [Source:HGNC:16191]
1525	CXADR	CXADR Ig-like cell adhesion molecule [Source:HGNC Symbol;Acc:HGNC:2559]
164312	LRRN4	leucine rich repeat neuronal 4 [Source:HGNC Symbol;Acc:HGNC:16208]
26022	TMEM98	transmembrane protein 98 [Source:HGNC Symbol;Acc:HGNC:24529]
2823	GPM6A	glycoprotein M6A [Source:HGNC Symbol;Acc:HGNC:4460]
284654	RSPO1	R-spondin 1 [Source:HGNC Symbol;Acc:HGNC:21679]
2938	GSTA1	glutathione S-transferase alpha 1 [Source:HGNC Symbol;Acc:HGNC:4626]
3855	KRT7	keratin 7 [Source:HGNC Symbol;Acc:HGNC:6445]
3861	KRT14	keratin 14 [Source:HGNC Symbol;Acc:HGNC:6416]
387496	RASL11A	RAS like family 11 member A [Source:HGNC Symbol;Acc:HGNC:23802]
387597	ILDR2	immunoglobulin like domain containing receptor 2 [Source:HGNC:18131]
389558	FAM180A	family with sequence similarity 180 member A [Source:HGNC:33773]
3911	LAMA5	laminin subunit alpha 5 [Source:HGNC Symbol;Acc:HGNC:6485]
3957	LGALS2	galectin 2 [Source:HGNC Symbol;Acc:HGNC:6562]
3995	FADS3	fatty acid desaturase 3 [Source:HGNC Symbol;Acc:HGNC:3576]
4642	MYO1D	myosin ID [Source:HGNC Symbol;Acc:HGNC:7598]
51196	PLCE1	phospholipase C epsilon 1 [Source:HGNC Symbol;Acc:HGNC:17175]
51232	CRIM1	cysteine rich transmembrane BMP regulator 1 [Source:HGNC:2359]
5349	FXYD3	FXYD domain containing ion transport regulator 3 [Source:HGNC:4027]
55512	SMPD3	sphingomyelin phosphodiesterase 3 [Source:HGNC Symbol;Acc:HGNC:14240]
57493	HEG1	heart development protein with EGF like domains 1 [Source:HGNC:29227]
5792	PTPRF	protein tyrosine phosphatase receptor type F [Source:HGNC:9670]
64759	TNS3	tensin 3 [Source:HGNC Symbol;Acc:HGNC:21616]
66000	TMEM108	transmembrane protein 108 [Source:HGNC Symbol;Acc:HGNC:28451]
6659	SOX4	SRY-box transcription factor 4 [Source:HGNC Symbol;Acc:HGNC:11200]
728113	ANXA8L1	annexin A8 like 1 [Source:HGNC Symbol;Acc:HGNC:23334]
7348	UPK1B	uroplakin 1B [Source:HGNC Symbol;Acc:HGNC:12578]
745	MYRF	myelin regulatory factor [Source:HGNC Symbol;Acc:HGNC:1181]
7490	WT1	WT1 transcription factor [Source:HGNC Symbol;Acc:HGNC:12796]
780	DDR1	discoidin domain receptor tyrosine kinase 1 [Source:HGNC Symbol;Acc:HGNC:2730]
80114	BICC1	BicC family RNA binding protein 1 [Source:HGNC Symbol;Acc:HGNC:19351]
9032	TM4SF5	transmembrane 4 L six family member 5 [Source:HGNC Symbol;Acc:HGNC:11857]
91584	PLXNA4	plexin A4 [Source:HGNC Symbol;Acc:HGNC:9102]
93035	PKHD1L1	PKHD1 like 1 [Source:HGNC Symbol;Acc:HGNC:20313]

Supplementary data

Table 2S Identification of EMT hallmark gene sets. Among the list, 11/55 non-tumor cell genes are involved in EMT pathway.

Gene Id	Gene Symbol	Gene Description (EMT genes are highlighted)
6678	SPARC	secreted protein acidic and cysteine rich [Source:HGNC Symbol;Acc:HGNC:11219]
5654	HTRA1	HtrA serine peptidase 1 [Source:HGNC Symbol;Acc:HGNC:9476]
1634	DCN	decorin [Source:HGNC Symbol;Acc:HGNC:2705]
633	BGN	biglycan [Source:HGNC Symbol;Acc:HGNC:1044]
51330	TNFRSF12A	TNF receptor superfamily member 12A [Source:HGNC Symbol;Acc:HGNC:18152]
7169	TPM2	tropomyosin 2 [Source:HGNC Symbol;Acc:HGNC:12011]
7052	TGM2	transglutaminase 2 [Source:HGNC Symbol;Acc:HGNC:11778]
2192	FBLN1	fibulin 1 [Source:HGNC Symbol;Acc:HGNC:3600]
5118	PCOLCE	procollagen C-endopeptidase enhancer [Source:HGNC Symbol;Acc:HGNC:8738]
59	ACTA2	"actin alpha 2, smooth muscle [Source:HGNC Symbol;Acc:HGNC:130]"
7168	TPM1	tropomyosin 1 [Source:HGNC Symbol;Acc:HGNC:12010]
1191	CLU	clusterin [Source:HGNC Symbol;Acc:HGNC:2095]
2934	GSN	gelsolin [Source:HGNC Symbol;Acc:HGNC:4620]
718	C3	complement C3 [Source:HGNC Symbol;Acc:HGNC:1318]
716	C1S	complement C1s [Source:HGNC Symbol;Acc:HGNC:1247]
710	SERPING1	serpin family G member 1 [Source:HGNC Symbol;Acc:HGNC:1228]
11098	PRSS23	serine protease 23 [Source:HGNC Symbol;Acc:HGNC:14370]
857	CAV1	caveolin 1 [Source:HGNC Symbol;Acc:HGNC:1527]
3005	H1-0	H1.0 linker histone [Source:HGNC Symbol;Acc:HGNC:4714]
3489	IGFBP6	insulin like growth factor binding protein 6 [Source:HGNC Symbol;Acc:HGNC:5475]
1410	CRYAB	crystallin alpha B [Source:HGNC Symbol;Acc:HGNC:2389]
165	AEBP1	AE binding protein 1 [Source:HGNC Symbol;Acc:HGNC:303]
3490	IGFBP7	insulin like growth factor binding protein 7 [Source:HGNC Symbol;Acc:HGNC:5476]
9806	SPOCK2	"SPARC (osteonectin), cwcv and kazal like domains proteoglycan 2 [Source:HGNC:13564]"
7167	TPI1	triosephosphate isomerase 1 [Source:HGNC Symbol;Acc:HGNC:12009]
6566	SLC16A1	solute carrier family 16 member 1 [Source:HGNC Symbol;Acc:HGNC:10922]
1363	CPE	carboxypeptidase E [Source:HGNC Symbol;Acc:HGNC:2303]
8611	PLPP1	phospholipid phosphatase 1 [Source:HGNC Symbol;Acc:HGNC:9228]
8848	TSC22D1	TSC22 domain family member 1 [Source:HGNC Symbol;Acc:HGNC:16826]
2202	EFEMP1	EGF containing fibulin extracellular matrix protein 1 [Source:HGNC:3218]
4781	NFIB	nuclear factor I B [Source:HGNC Symbol;Acc:HGNC:7785]
23710	GABARAPL1	GABA type A receptor associated protein like 1 [Source:HGNC Symbol;Acc:HGNC:4068]
9411	ARHGAP29	Rho GTPase activating protein 29 [Source:HGNC Symbol;Acc:HGNC:30207]
977	CD151	CD151 molecule (Raph blood group) [Source:HGNC Symbol;Acc:HGNC:1630]
55107	ANO1	anoctamin 1 [Source:HGNC Symbol;Acc:HGNC:21625]
481	ATP1B1	ATPase Na+/K+ transporting subunit beta 1 [Source:HGNC Symbol;Acc:HGNC:804]
4734	NEDD4	NEDD4 E3 ubiquitin protein ligase [Source:HGNC Symbol;Acc:HGNC:7727]
7103	TSPAN8	tetraspanin 8 [Source:HGNC Symbol;Acc:HGNC:11855]
100532736	MICOS10-NBL1	MICOS10-NBL1 readthrough [Source:HGNC Symbol;Acc:HGNC:48338]
10079	ATP9A	ATPase phospholipid transporting 9A (putative) [Source:HGNC:13540]
10787	NCKAP1	NCK associated protein 1 [Source:HGNC Symbol;Acc:HGNC:7666]
151887	CCDC80	coiled-coil domain containing 80 [Source:HGNC Symbol;Acc:HGNC:30649]
23164	MPRIP	myosin phosphatase Rho interacting protein [Source:HGNC Symbol;Acc:HGNC:30321]
23213	SULF1	sulfatase 1 [Source:HGNC Symbol;Acc:HGNC:20391]
3281	HSBP1	heat shock factor binding protein 1 [Source:HGNC Symbol;Acc:HGNC:5203]
379	ARL4D	ADP ribosylation factor like GTPase 4D [Source:HGNC Symbol;Acc:HGNC:656]
3791	KDR	kinase insert domain receptor [Source:HGNC Symbol;Acc:HGNC:6307]
4054	LTBP3	latent transforming growth factor beta binding protein 3 [Source:HGNC:6716]
51765	STK26	serine/threonine kinase 26 [Source:HGNC Symbol;Acc:HGNC:18174]
55353	LAPTM4B	lysosomal protein transmembrane 4 beta [Source:HGNC Symbol;Acc:HGNC:13646]
58189	WFDC1	WAP four-disulfide core domain 1 [Source:HGNC Symbol;Acc:HGNC:15466]
58480	RHOU	ras homolog family member U [Source:HGNC Symbol;Acc:HGNC:17794]
6038	RNASE4	ribonuclease A family member 4 [Source:HGNC Symbol;Acc:HGNC:10047]
8510	MMP23B	matrix metallopeptidase 23B [Source:HGNC Symbol;Acc:HGNC:7171]

Supplementary data

Figure 3S Network of OMICS profile of interest. Significant correlation between gene of interest (RNA expression) in TCGA cohort MESO with OMICS profile with FDR < 0.05. OMICS includes mRNA, miRNA, copy number alteration (CNA), methylation, and reverse phase protein analysis (RPPA) correlation was also analyzed. Only the most significantly correlated genes (*COL5A2*, *ITGAV*, *SPARC* and *ACTA2*) were presented.

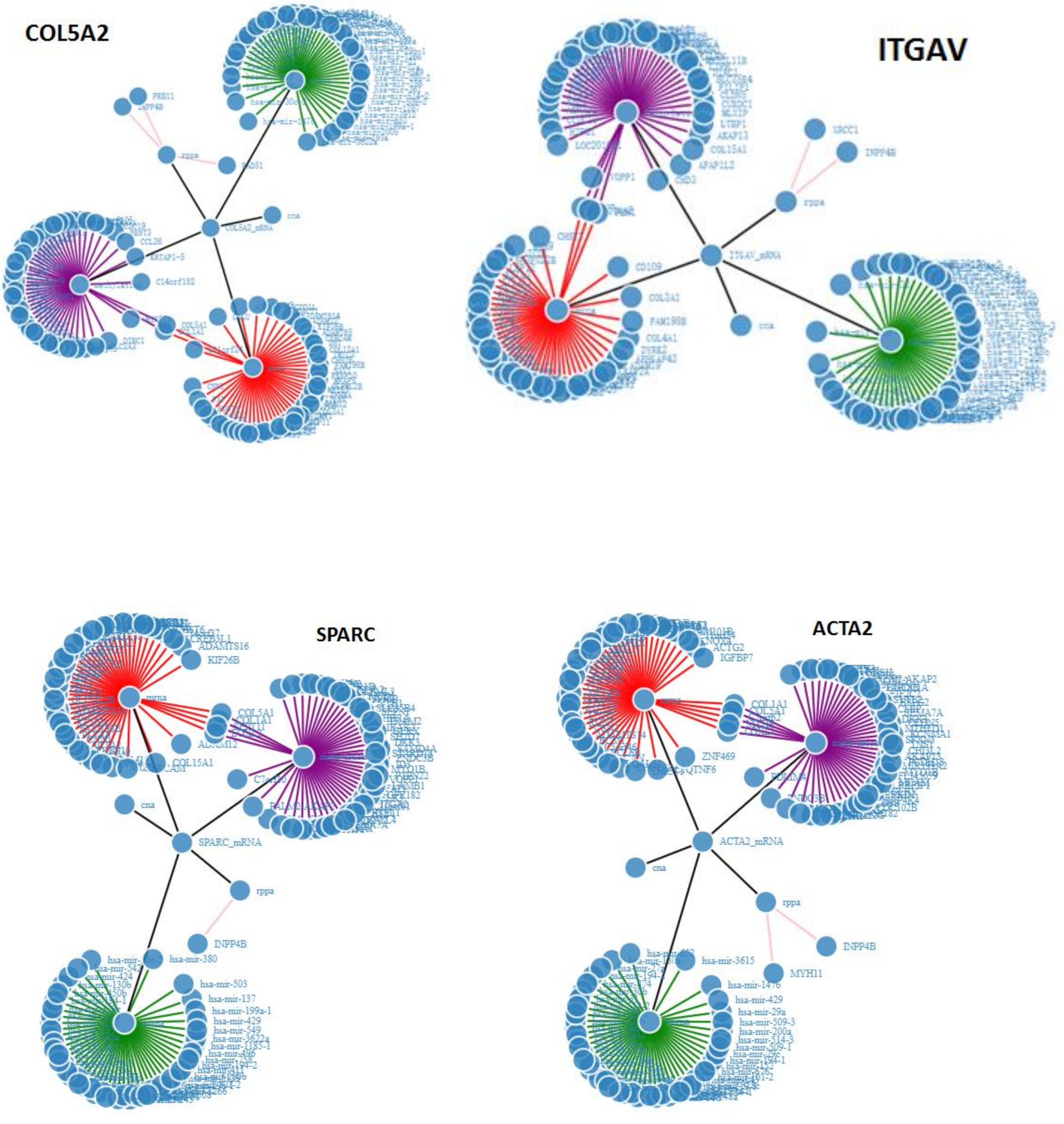

Supplementary data

Fig. 4S Gene expression *COL5A2, ITGAV, SPARC* and *ACTA2* in Epithelioid *vs* non-Epithelioid subtype in MESO patients. A) Dotplot of 4 gene expression in scRNA-Seq was shown in five naïve MPM patients, and CDH1 and VIM were included as controls. B) Histology of HE staining of biphasic (SMTR02T0) and epithelioid (SMTR03T0) subtype.

A

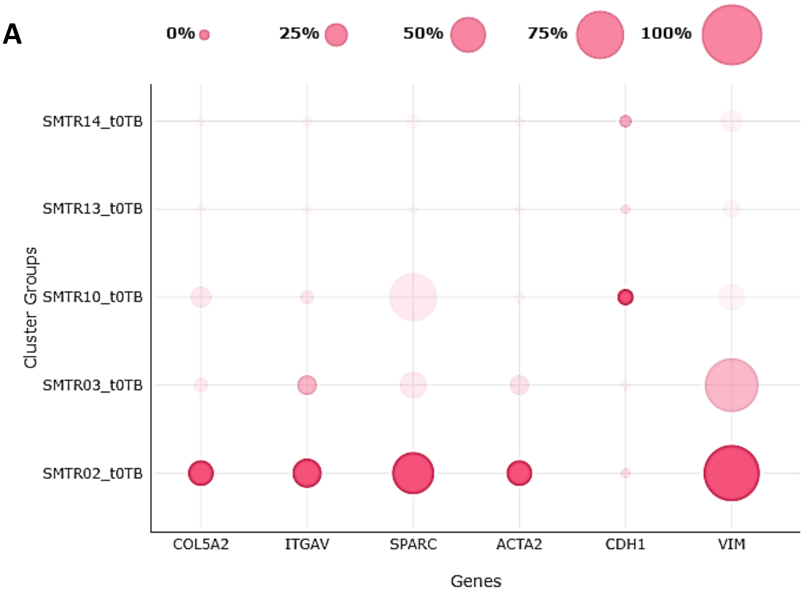

B

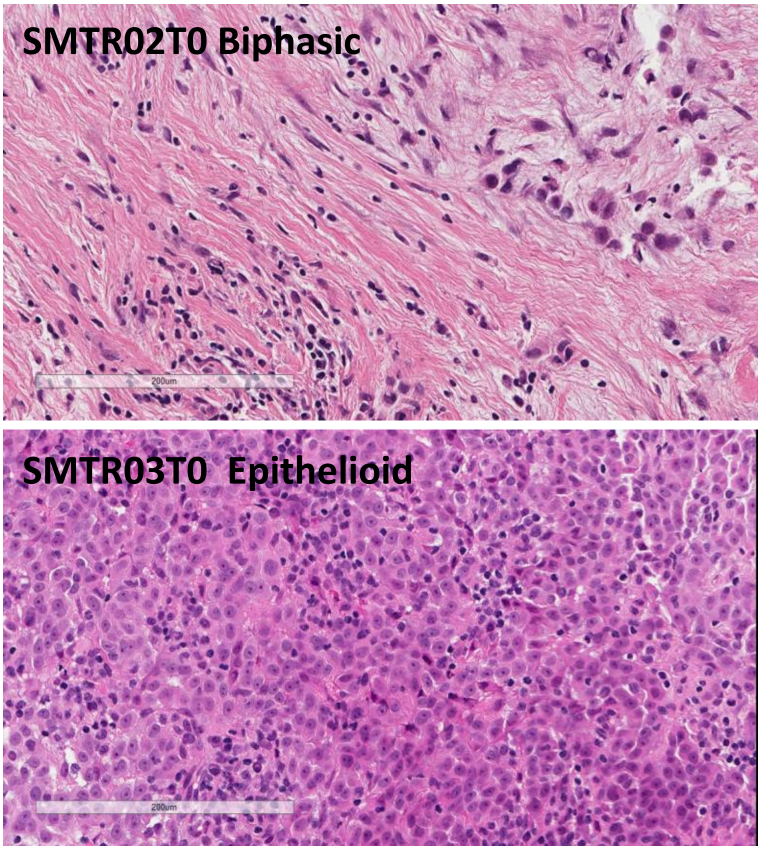

Supplementary data

Figure 5S *COL5A2* and *SPARC* gene expression correlated with immune enrichment in MESO cohort of TCGA database. COL5A2 (top panel) and SPARC (bottom panel) gene expression correlated with immune enrichment: Macrophage, Th2, Treg and NKT in TCGA.

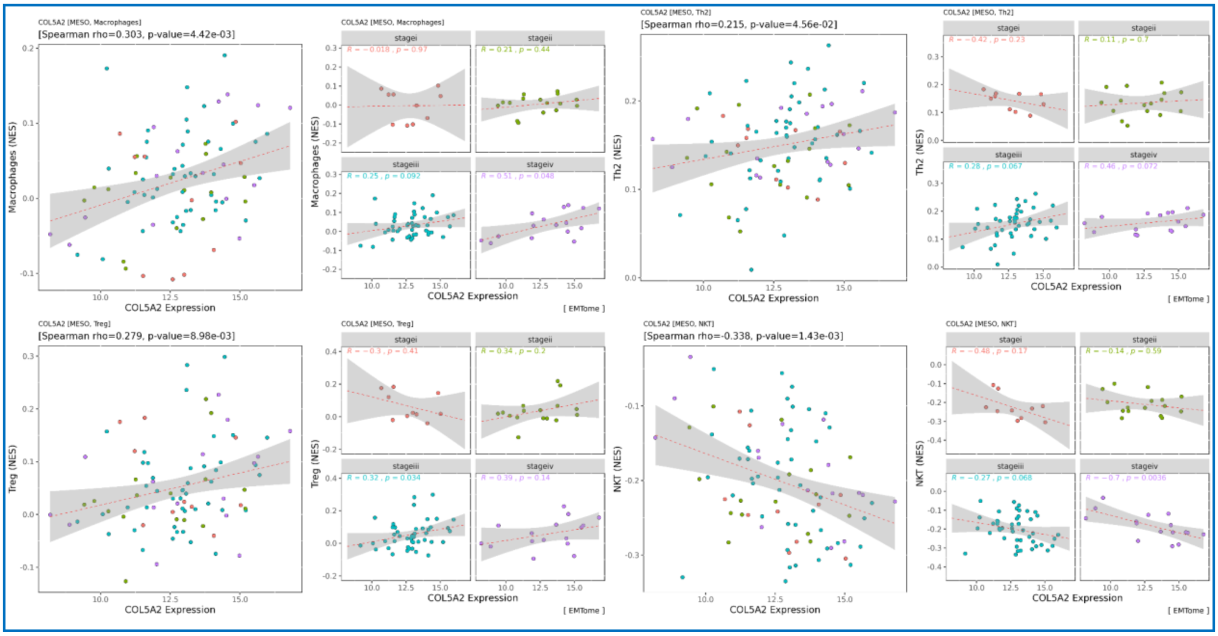

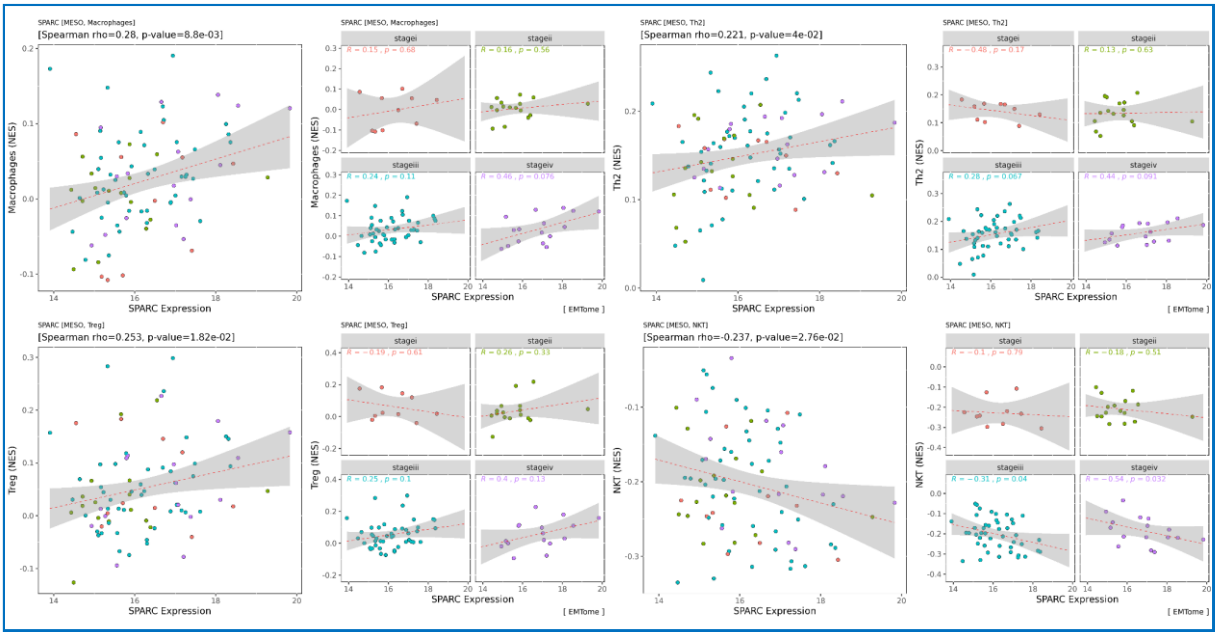

Supplementary data

Figure 6S *ITGAV* and *ACTA2* gene expression correlated with immune enrichment in MESO cohort of TCGA database. *ITGAV* (top panel) and *ACTA2* (bottom) gene expression in correlation with Macrophage, Th2, Treg and NKT in TCGA.

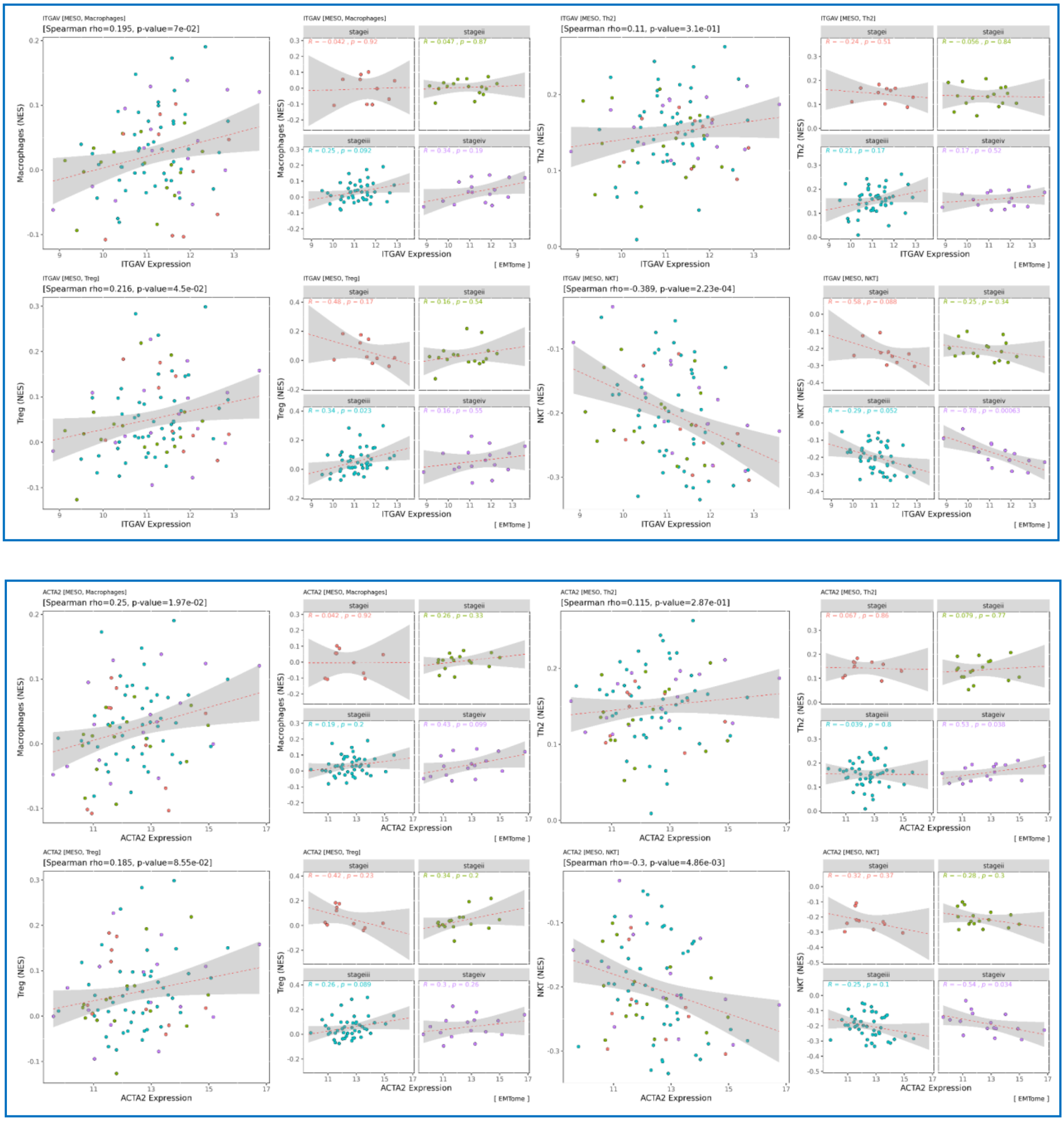

Supplementary data

Figure 7S Gene expression correlated with immune enrichment in MESO cohort of TCGA database. *ITGAV* (A) and *ACTA2* (B)

A

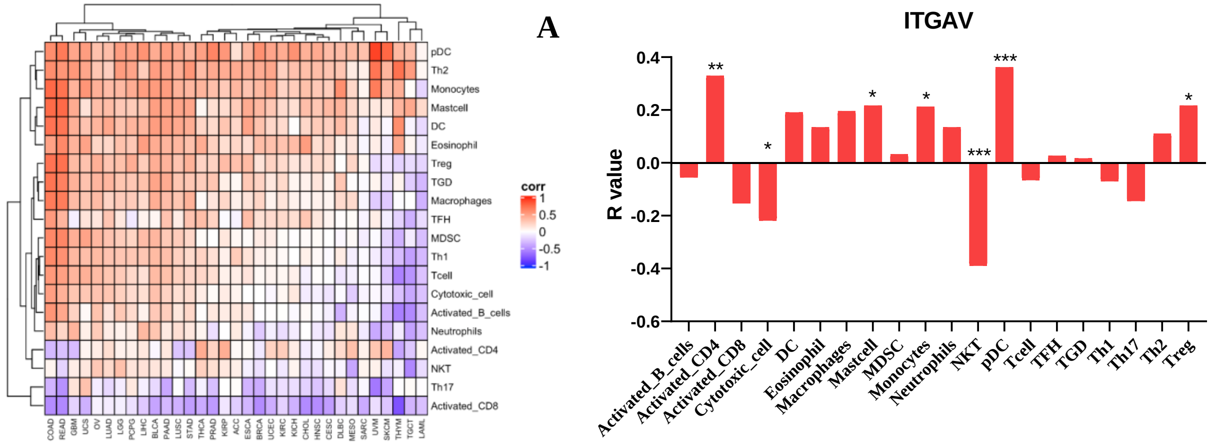

B

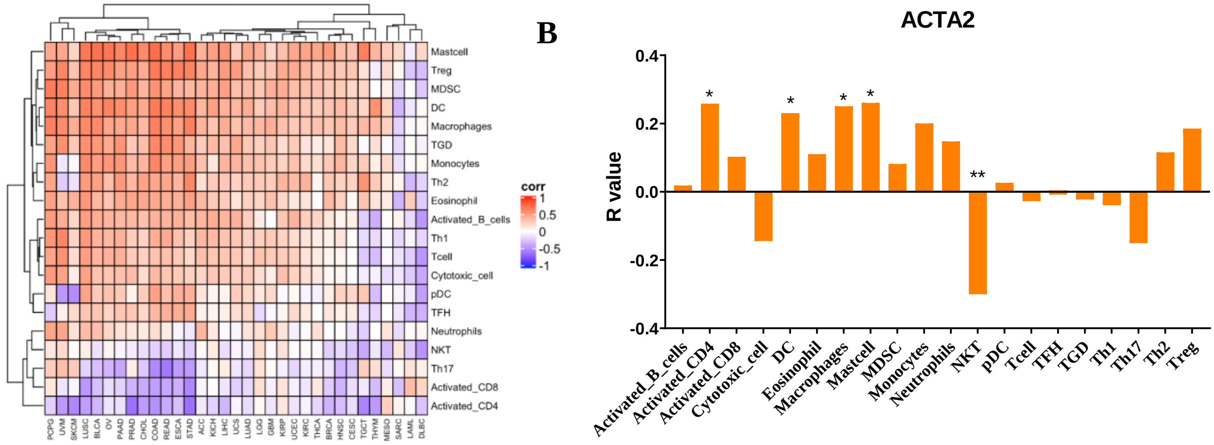

Supplementary data

Figure 8S The immune infiltration associated with gene expression and clinical outcome in MESO of TCGA.

Gene module performed the correlation of gene expression with cancer associated fibroblasts (CAF) and immune cell infiltrates MDSC, Th2 cells in MESO.

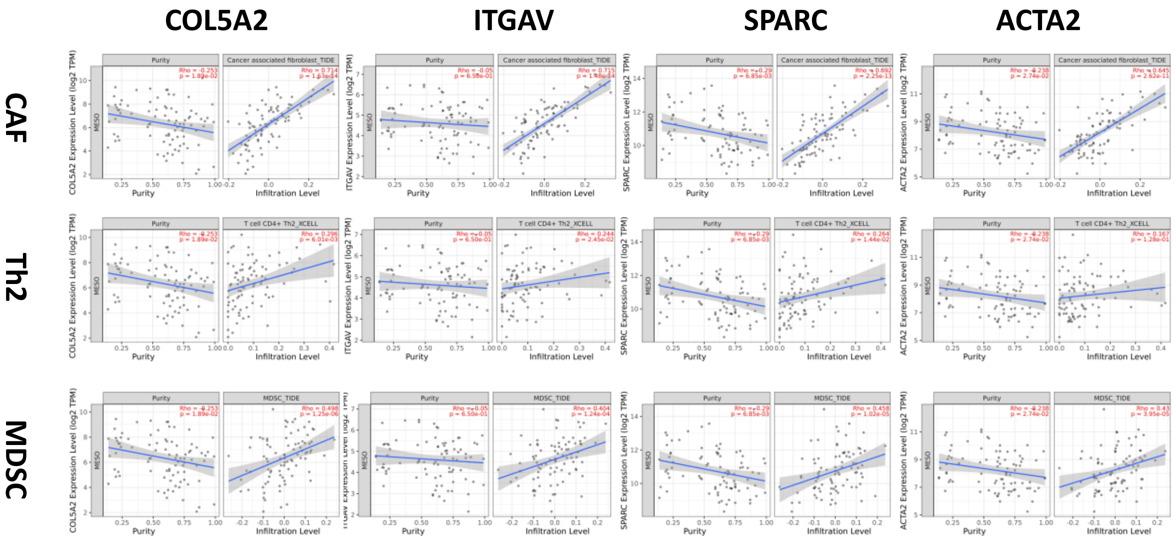

Supplementary data

Figure 9S The immune infiltration associated with gene expression and clinical outcome in MESO of TCGA.

Gene module performed the correlation of gene expression with immune cell infiltration level in MESO. EMT gene expression and immune infiltrates of M2 macrophage and Treg cells in correlation with clinical outcome in MESO cohort.

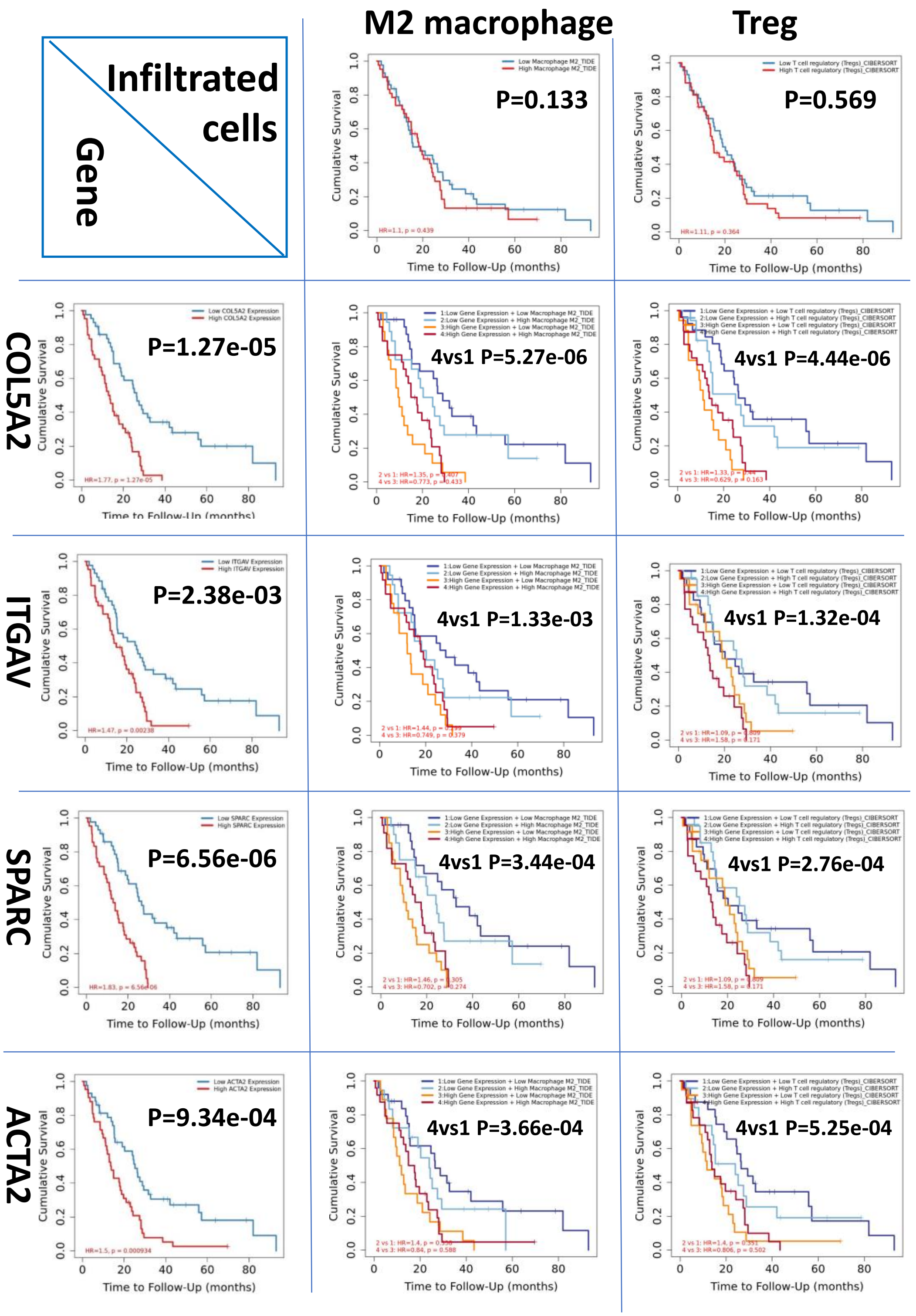

Supplement: Supplementary file 1 — Supplementary Information. [file 41598_2022_4973_MOESM1_ESM.pdf]
